# Supplementary figures and images for: The Genetic Architecture of Adaptations to High Altitude in Ethiopia
Source: PLoS Genet. 2012 Dec 6;8(12):e1003110. doi: 10.1371/journal.pgen.1003110 (PMC3516565; doi:10.1371/journal.pgen.1003110)

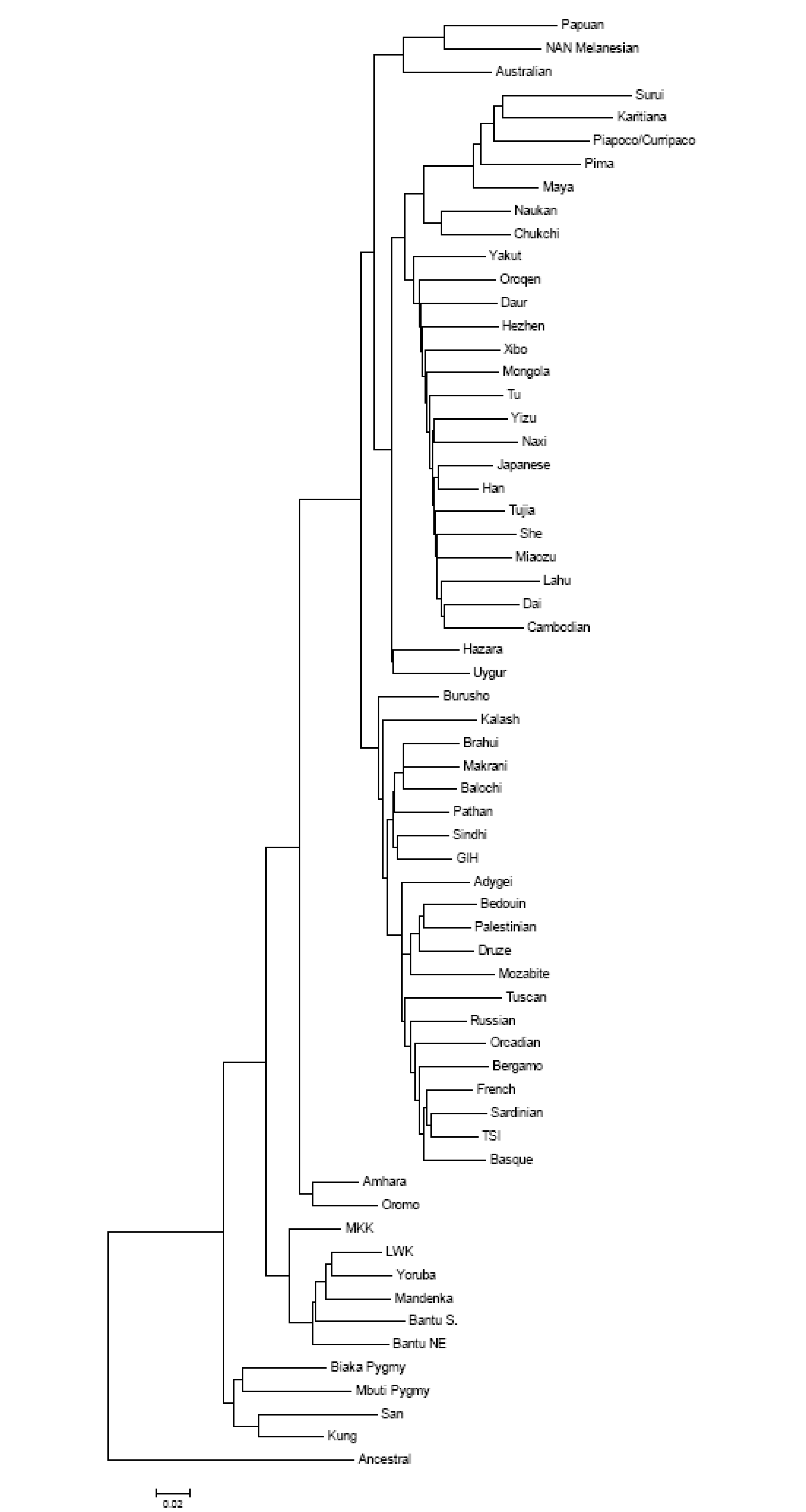

Supplement: Figure S1 — FST-based neighbor-joining tree showing the relationships of the Ethiopians to the worldwide populations. Oromo and Amhara cluster closely together in the tree and occupy an intermediate position between African (with the exception of the Mozabites) and non-African populations. An ancestral population fixed for ancestral alleles at all SNPs was used. (TIF) [file pgen.1003110.s001.tif]

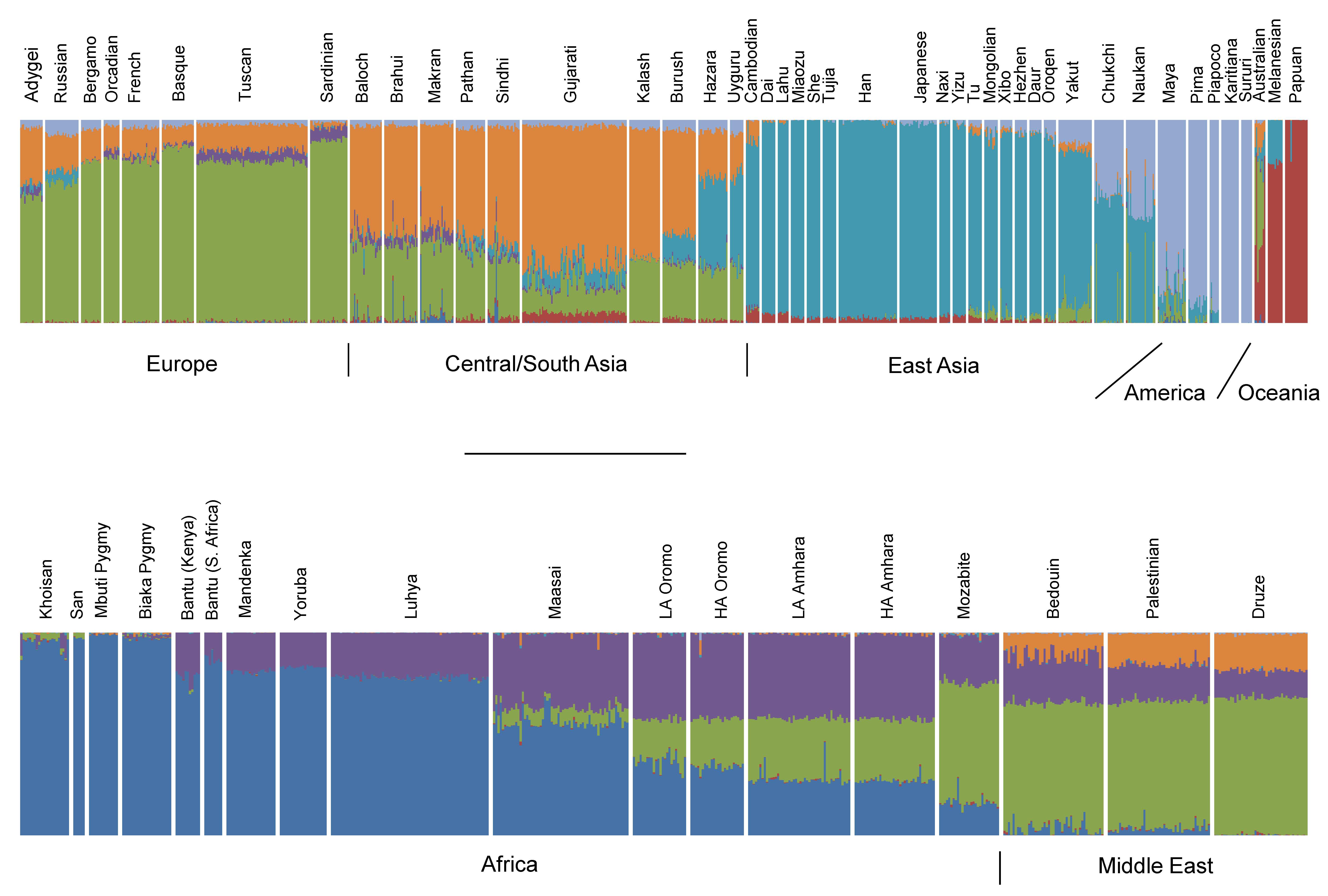

Supplement: Figure S2 — Worldwide STRUCTURE plot. Each vertical line represents an individual, and the colors comprising each line correspond to the inferred proportion of ancestry from seven ancestral populations using 57652 random autosomal SNPs. (TIF) [file pgen.1003110.s002.tif]

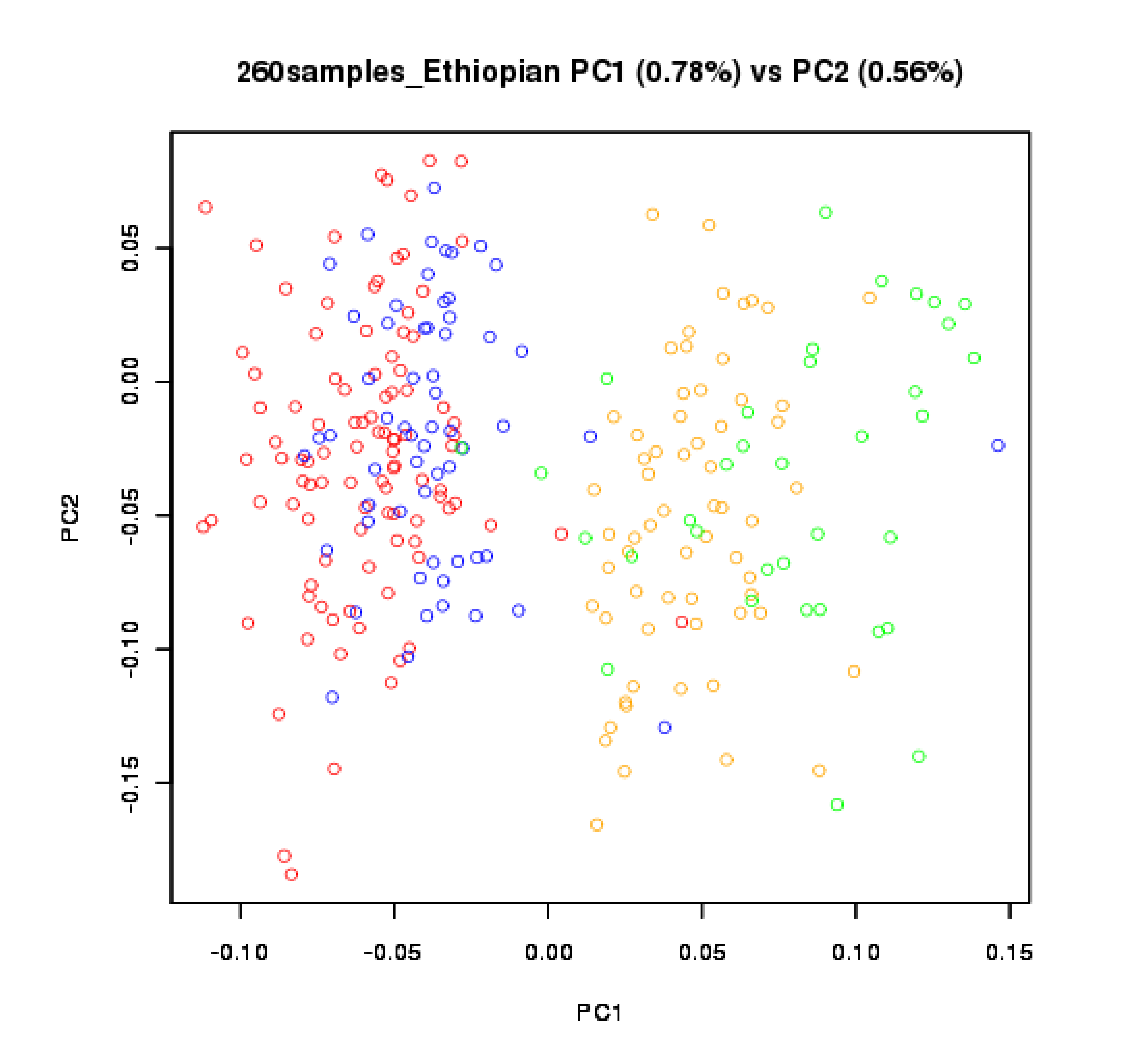

Supplement: Figure S3 — Scatter plot of the first two coordinates obtained by principal component analysis. A set of 13,000 random autosomal SNPs were used. HA Amhara individuals are represented in red, LA Amhara in blue, HA Oromo in orange and LA Oromo in green. (TIF) [file pgen.1003110.s003.tif]

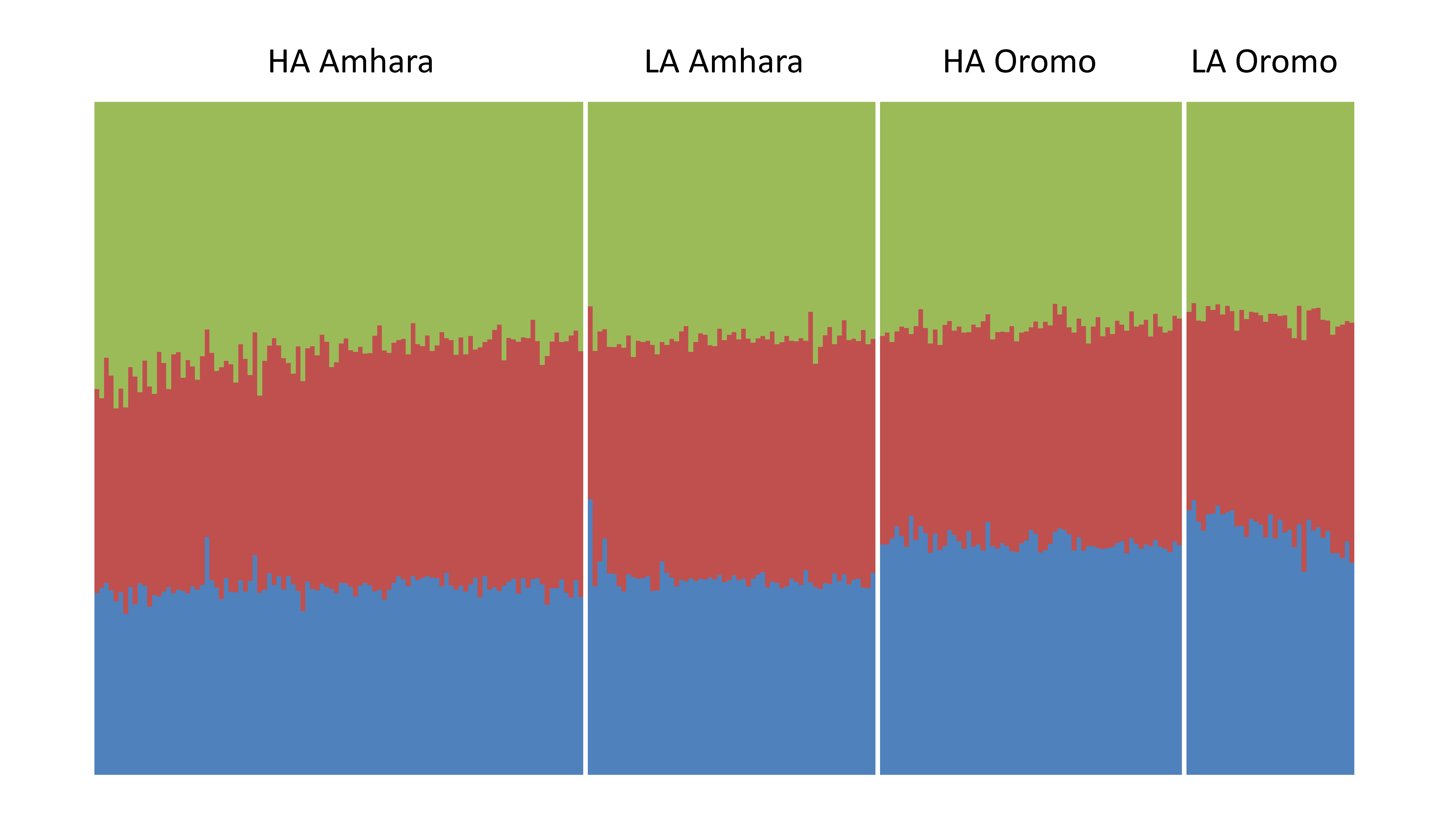

Supplement: Figure S4 — Ethiopian STRUCTURE plot. Each vertical line represents an individual, and the colors comprising each line correspond to the inferred proportion of ancestry from three ancestral populations using 57652 random autosomal SNPs. Samples were ordered from left to right as follows: HA Amhara, LA Amhara, HA Oromo and LA Oromo. (TIF) [file pgen.1003110.s004.tif]

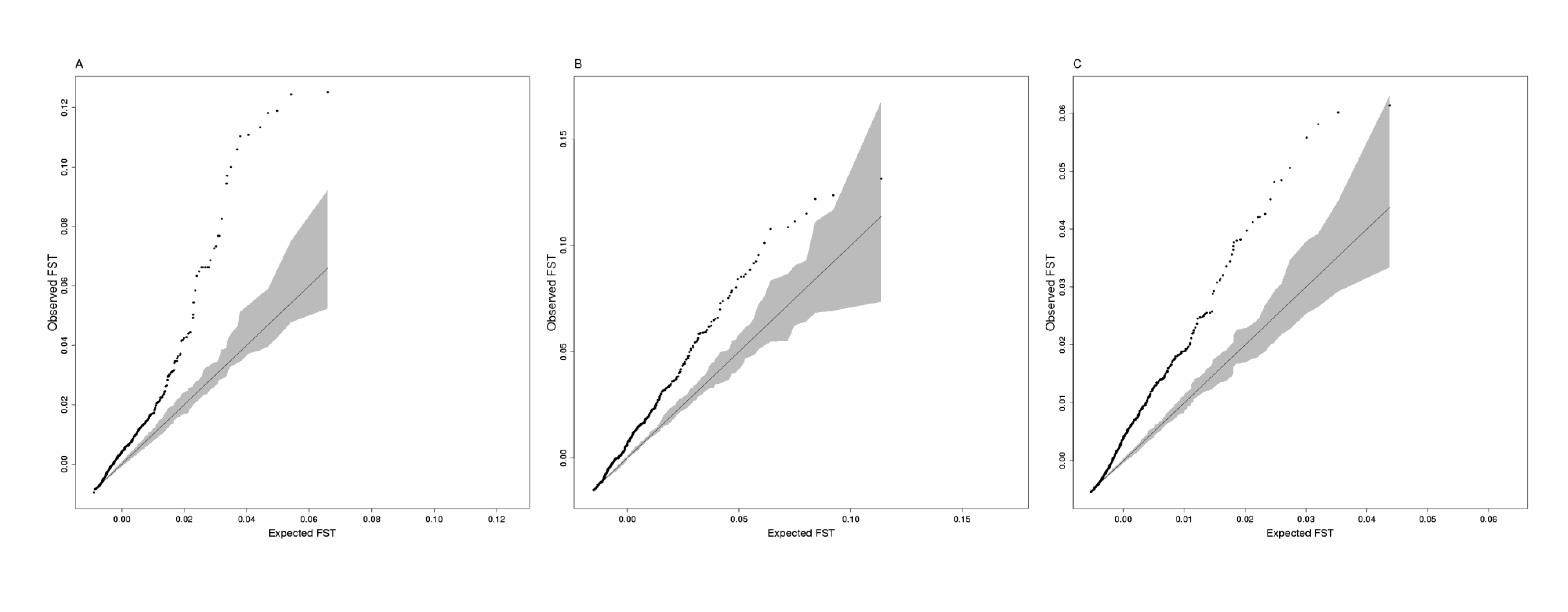

Supplement: Figure S5 — Population differentiation among Ethiopian subgroups. The QQplots represent difference in allele frequency as summarized by FST between HA and LA Amhara (A), Oromo (B) and Ethiopia (C). The observed FST distribution is ranked from smallest to largest and plotted against the expected FST in black. The expected FST distribution was obtained by permuting the subgroup labels mimicking random mating. The grey area indicates the 95% confidence interval of the expected distribution (see Methods). (TIF) [file pgen.1003110.s005.tif]

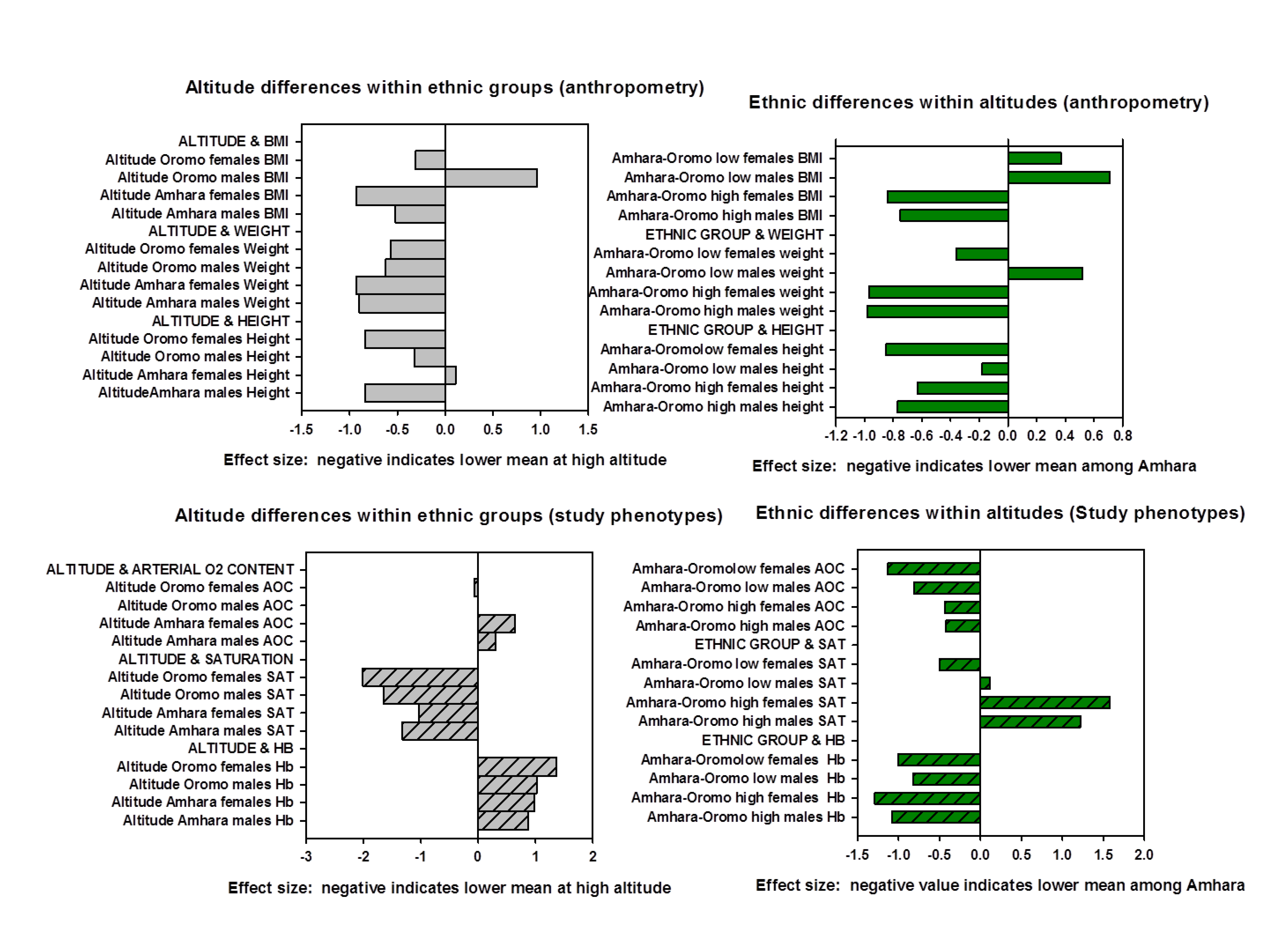

Supplement: Figure S6 — Altitude differences in anthropometric and phenotypic characteristics are summarized in the left two (gray) panels on terms of effect size d. Ethnic differences are summarized in the right two (green) panels the same way. D is dimensionless and is calculated as the difference between two sample means divided by their pooled standard deviation. Comparison based on d values allows contrasting the altitude and ethnic-group differences in phenotypes independent of the units of measurement. By convention effect sizes of 0.8 or more are considered to be ‘large’ [82]. (TIF) [file pgen.1003110.s006.tif]

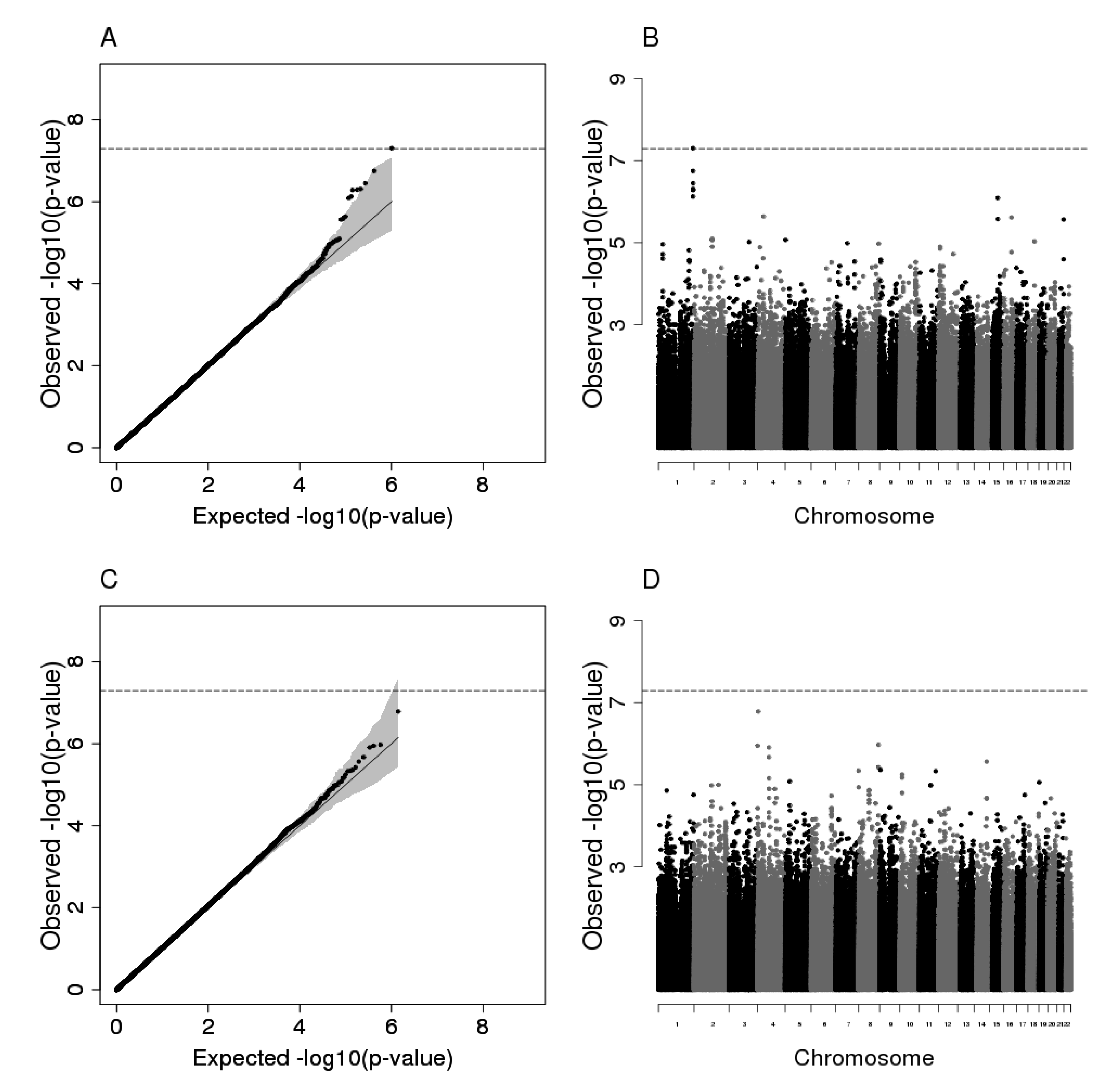

Supplement: Figure S7 — Amhara Hb level and O2 sat GWAS results. The QQplot compares the observed −log10 association p-value distribution (y-axis) with an expected distribution (x-axis) in black (see Methods) for Hb (A) and O2 sat (C). The grey area represents the 95% confidence interval. The Manhattan plot shows the observed −log10 association p-value of SNPs for Hb (B) and O2 sat (D). (TIF) [file pgen.1003110.s007.tif]

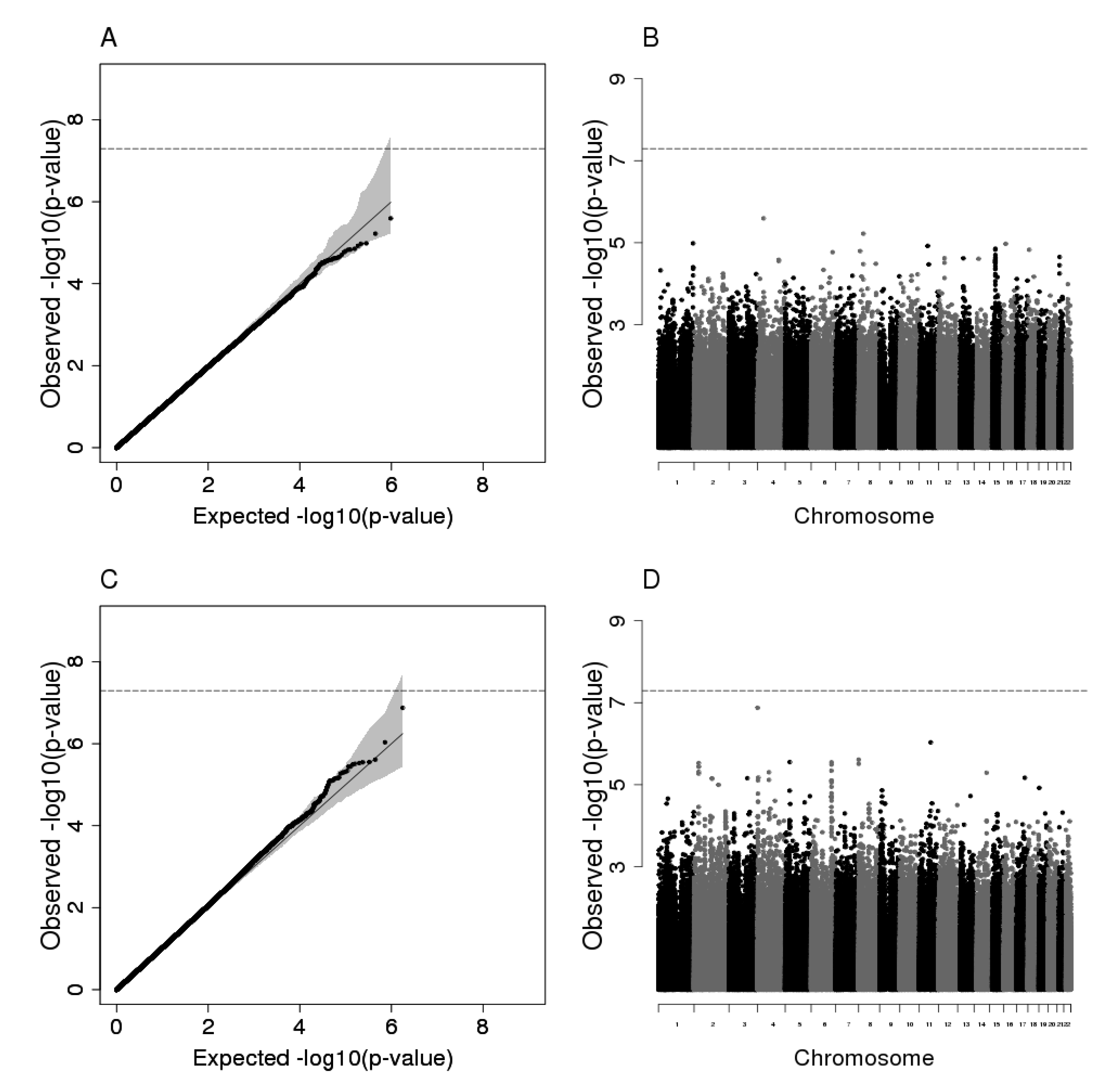

Supplement: Figure S8 — HA Amhara Hb level and O2 sat GWAS results. The QQplot compares the observed −log10 association p-value distribution (y-axis) with an expected distribution (x-axis) in black (see Methods) for Hb (A) and O2 sat (C). The grey area represents the 95% confidence interval. The Manhattan plot shows the observed −log10 association p-value of SNPs for Hb (B) and O2 sat (D). (TIF) [file pgen.1003110.s008.tif]

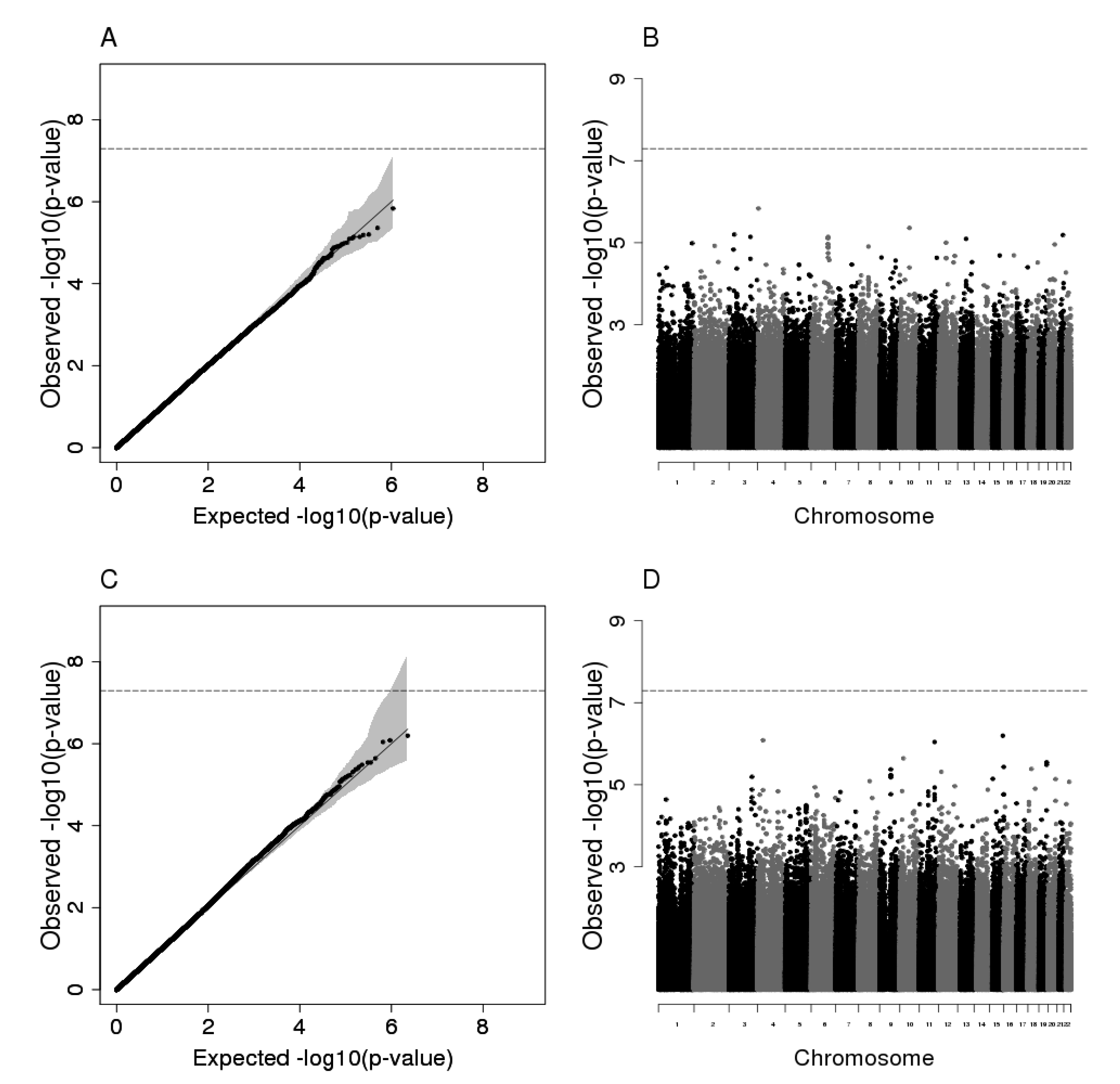

Supplement: Figure S9 — LA Amhara Hb level and O2 sat GWAS results. The QQplot compares the observed −log10 association p-value distribution (y-axis) with an expected distribution (x-axis) in black (see Methods) for Hb (A) and O2 sat (C). The grey area represents the 95% confidence interval. The Manhattan plot shows the observed −log10 association p-value of SNPs for Hb (B) and O2 sat (D). (TIF) [file pgen.1003110.s009.tif]

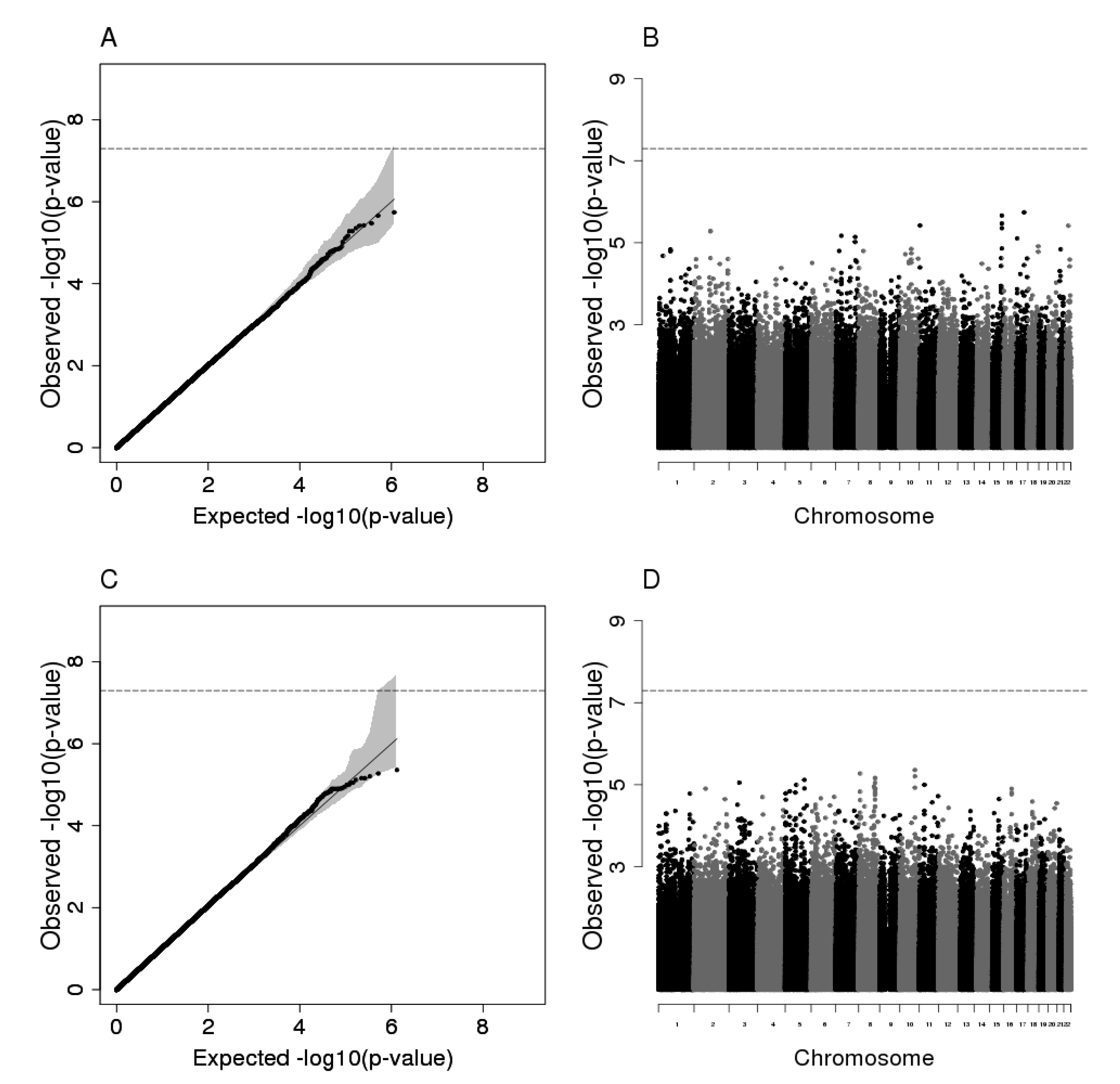

Supplement: Figure S10 — Oromo Hb level and O2 sat GWAS results. The QQplot compares the observed −log10 association p-value distribution (y-axis) with an expected distribution (x-axis) in black (see Methods) for Hb (A) and O2 sat (C). The grey area represents the 95% confidence interval. The Manhattan plot shows the observed −log10 association p-value of SNPs for Hb (B) and O2 sat (D). (TIF) [file pgen.1003110.s010.tif]

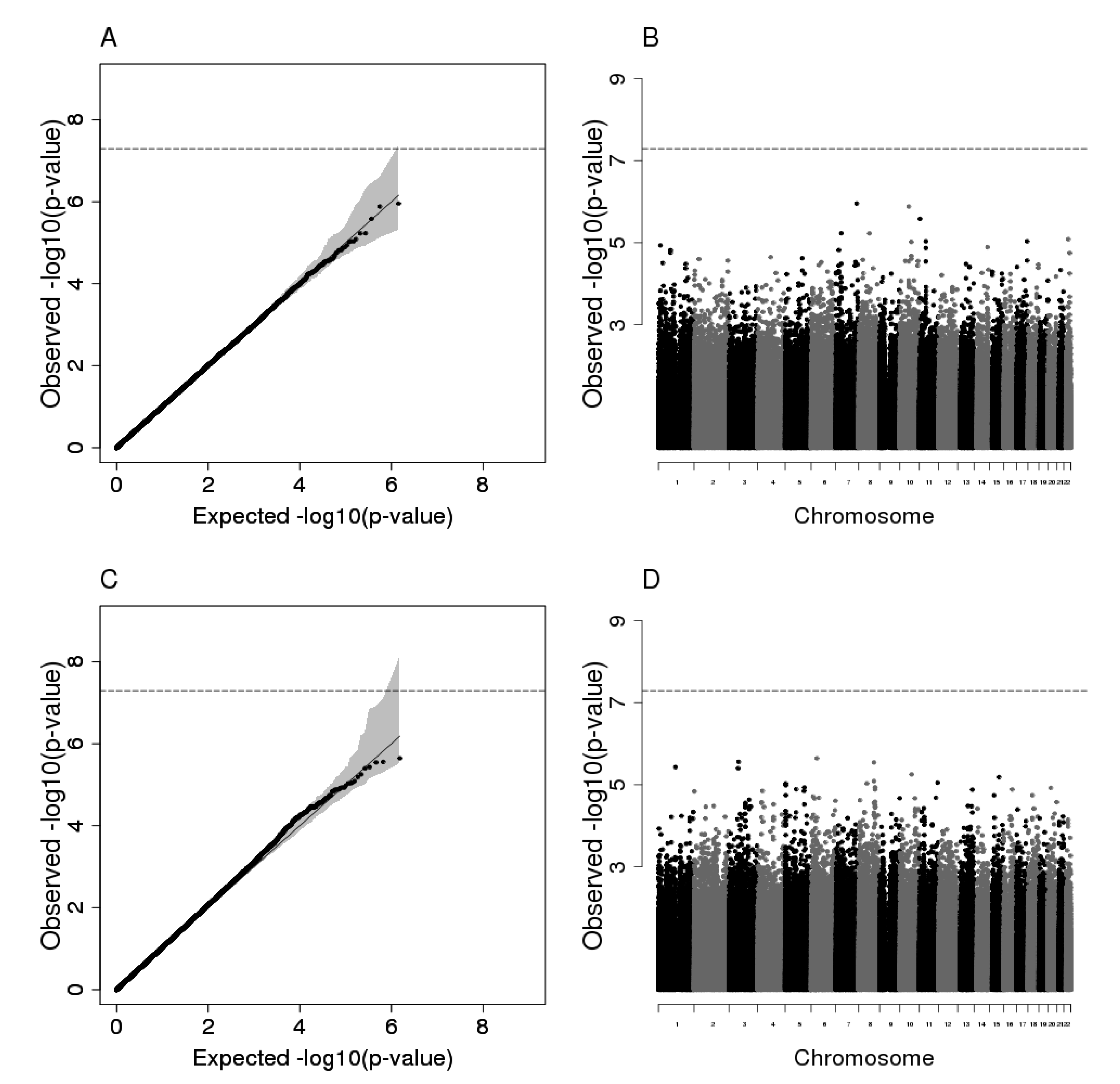

Supplement: Figure S11 — HA Oromo Hb level and O2 sat GWAS results. The QQplot compares the observed −log10 association p-value distribution (y-axis) with an expected distribution (x-axis) in black (see Methods) for Hb (A) and O2 sat (C). The grey area represents the 95% confidence interval. The Manhattan plot shows the observed −log10 association p-value of SNPs for Hb (B) and O2 sat (D). (TIF) [file pgen.1003110.s011.tif]

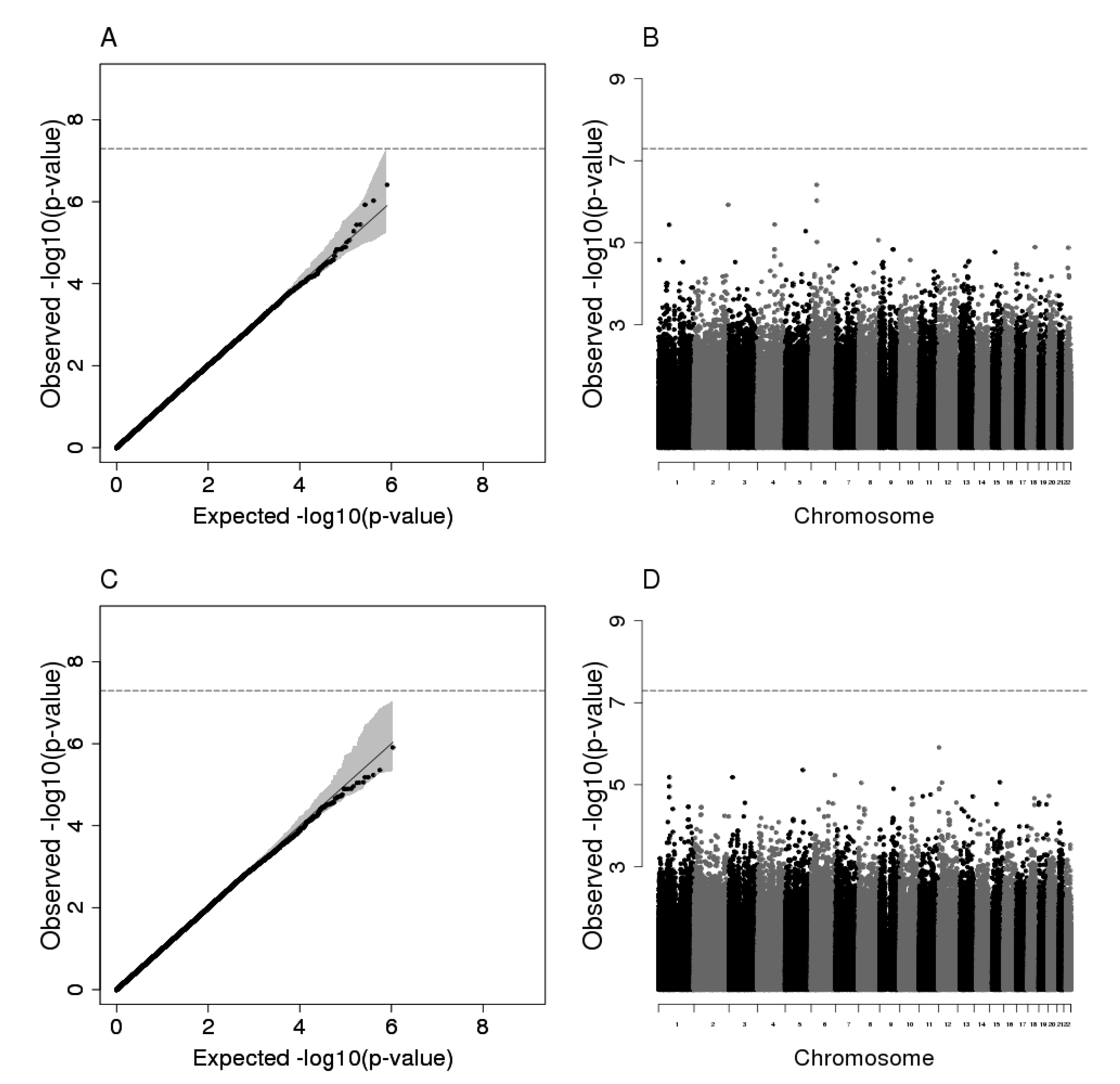

Supplement: Figure S12 — LA Oromo Hb level and O2 sat GWAS results. The QQplot compares the observed −log10 association p-value distribution (y-axis) with an expected distribution (x-axis) in black (see Methods) for Hb (A) and O2 sat (C). The grey area represents the 95% confidence interval. The Manhattan plot shows the observed −log10 association p-value of SNPs for Hb (B) and O2 sat (D). (TIF) [file pgen.1003110.s012.tif]

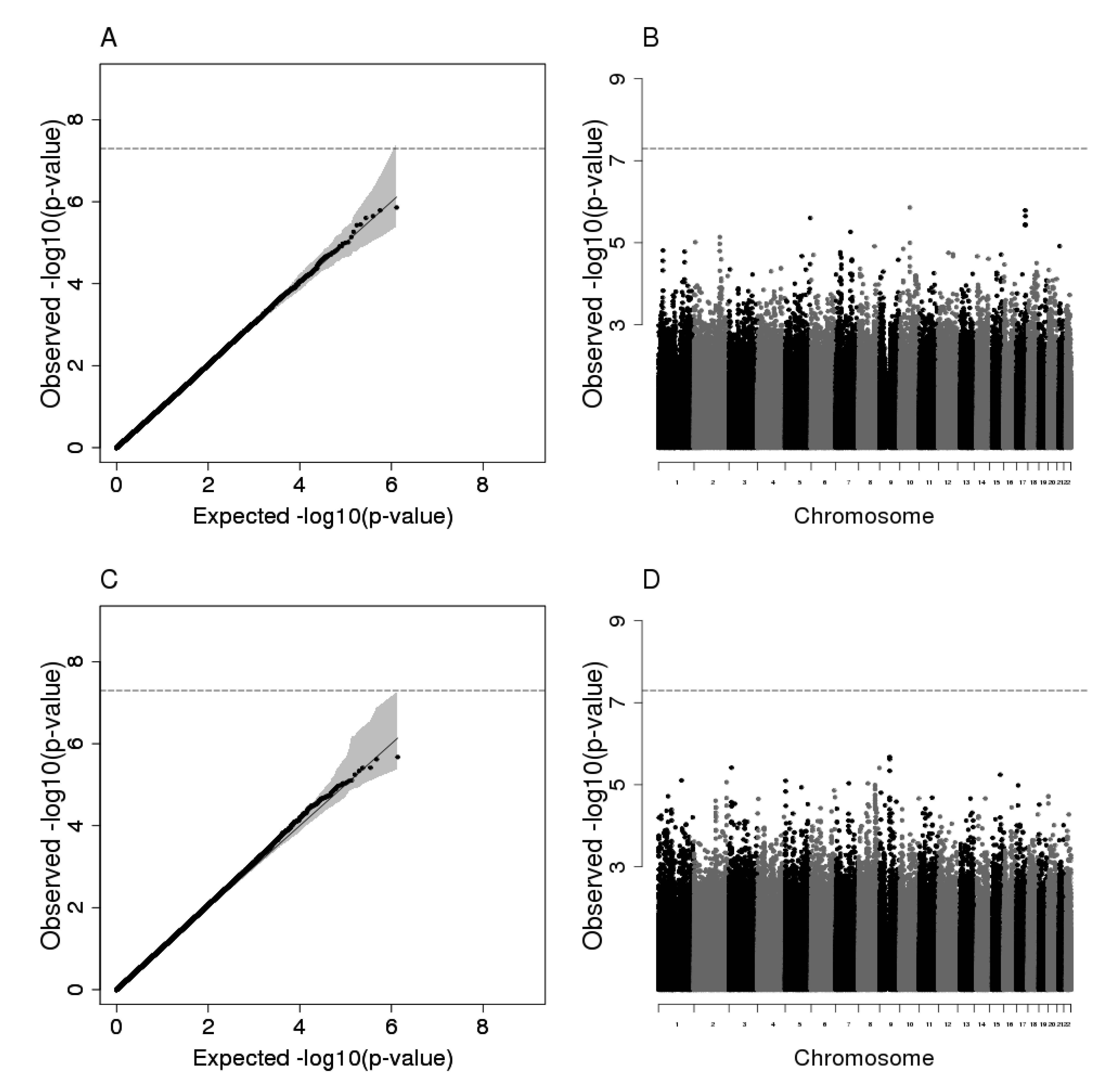

Supplement: Figure S13 — Ethiopia Hb level and O2 sat GWAS results. The QQplot compares the observed −log10 association p-value distribution (y-axis) with an expected distribution (x-axis) in black (see Methods) for Hb (A) and O2 sat (C). The grey area represents the 95% confidence interval. The Manhattan plot shows the observed −log10 association p-value of SNPs for Hb (B) and O2 sat (D). (TIF) [file pgen.1003110.s013.tif]

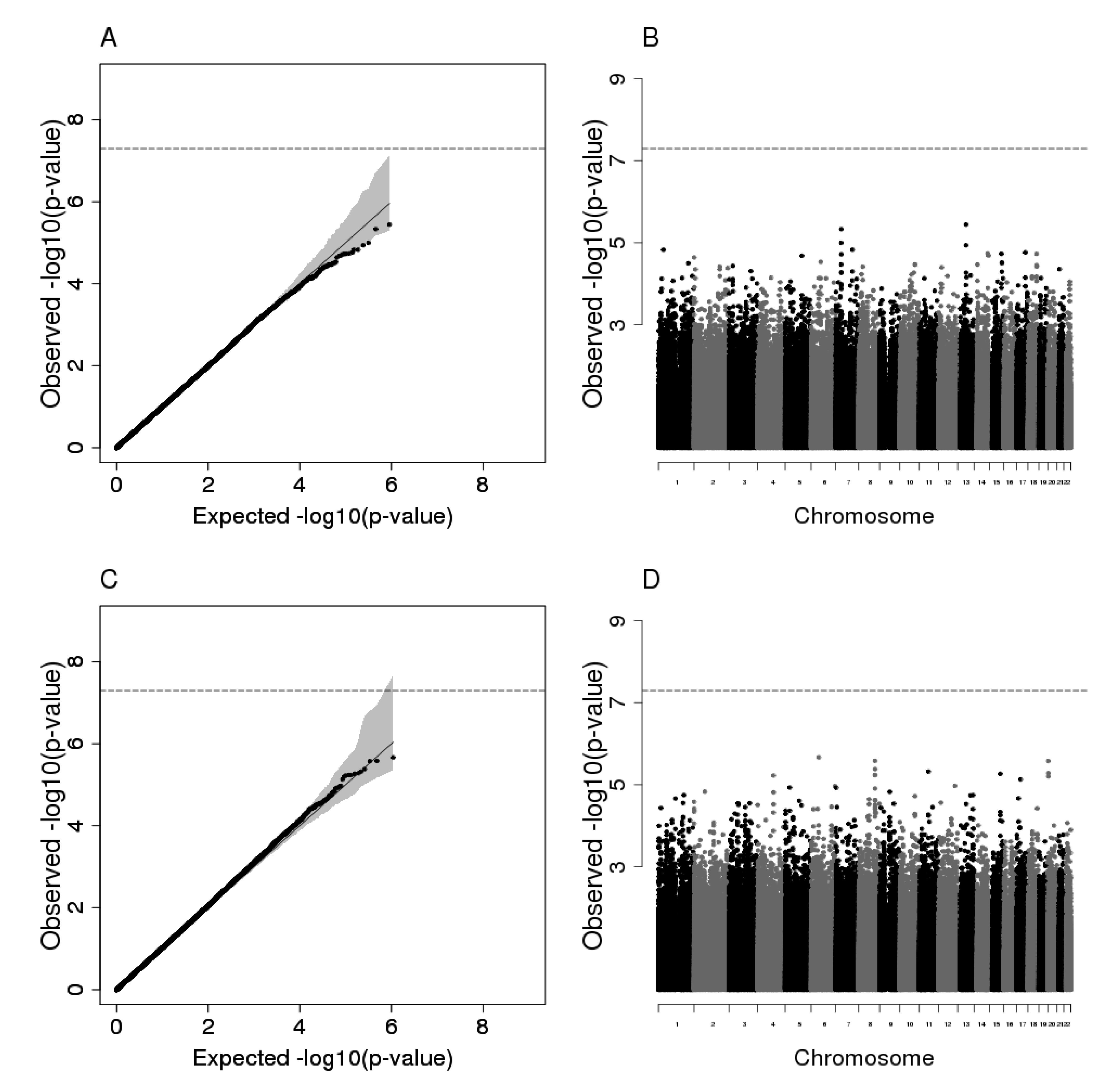

Supplement: Figure S14 — HA Ethiopia Hb level and O2 sat GWAS results. The QQplot compares the observed −log10 association p-value distribution (y-axis) with an expected distribution (x-axis) in black (see Methods) for Hb (A) and O2 sat (C). The grey area represents the 95% confidence interval. The Manhattan plot shows the observed −log10 association p-value of SNPs for Hb (B) and O2 sat (D). (TIF) [file pgen.1003110.s014.tif]

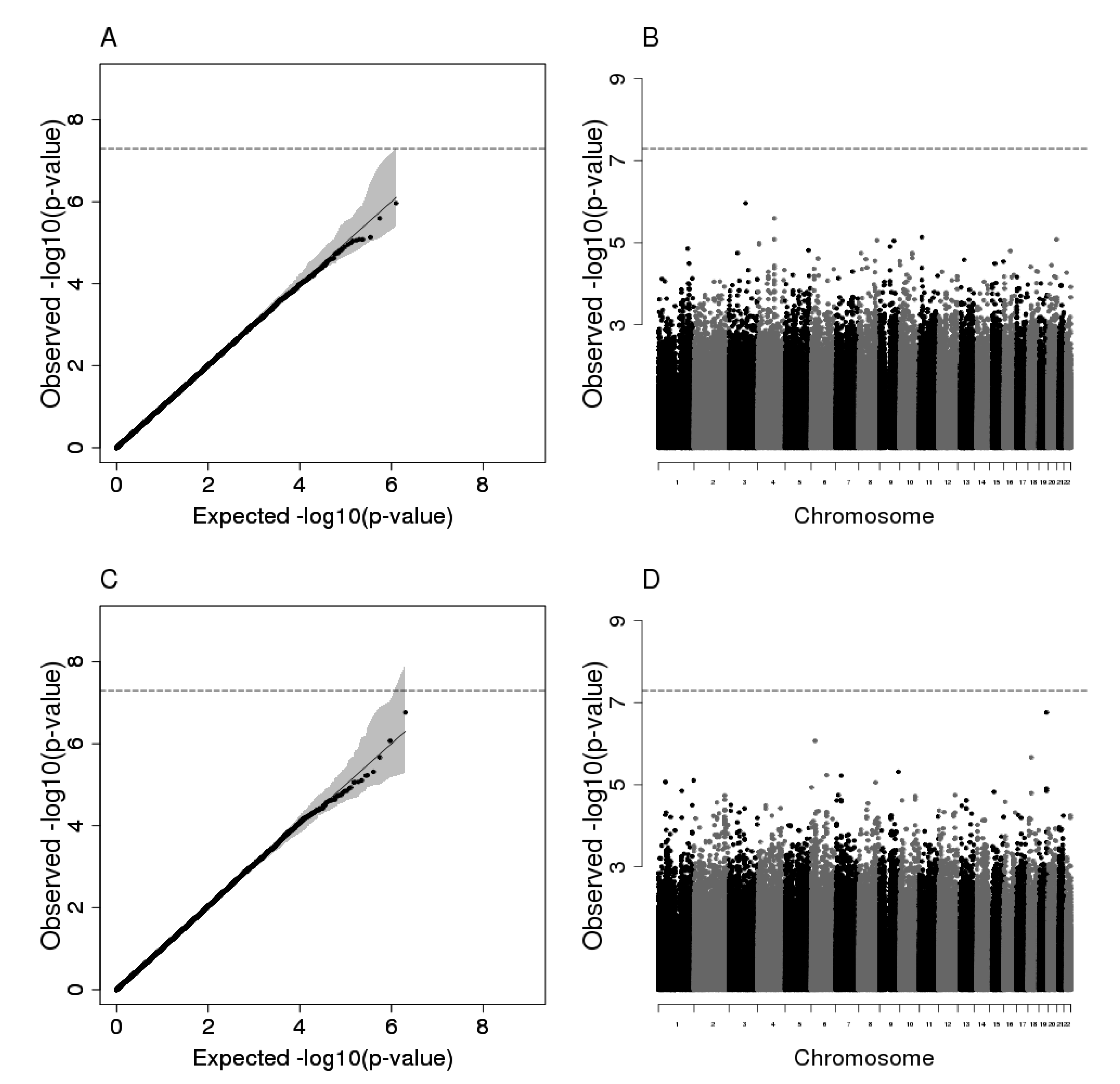

Supplement: Figure S15 — LA Ethiopia Hb level and O2 sat GWAS results. The QQplot compares the observed −log10 association p-value distribution (y-axis) with an expected distribution (x-axis) in black (see Methods) for Hb (A) and O2 sat (C). The grey area represents the 95% confidence interval. The Manhattan plot shows the observed −log10 association p-value of SNPs for Hb (B) and O2 sat (D). (TIF) [file pgen.1003110.s015.tif]

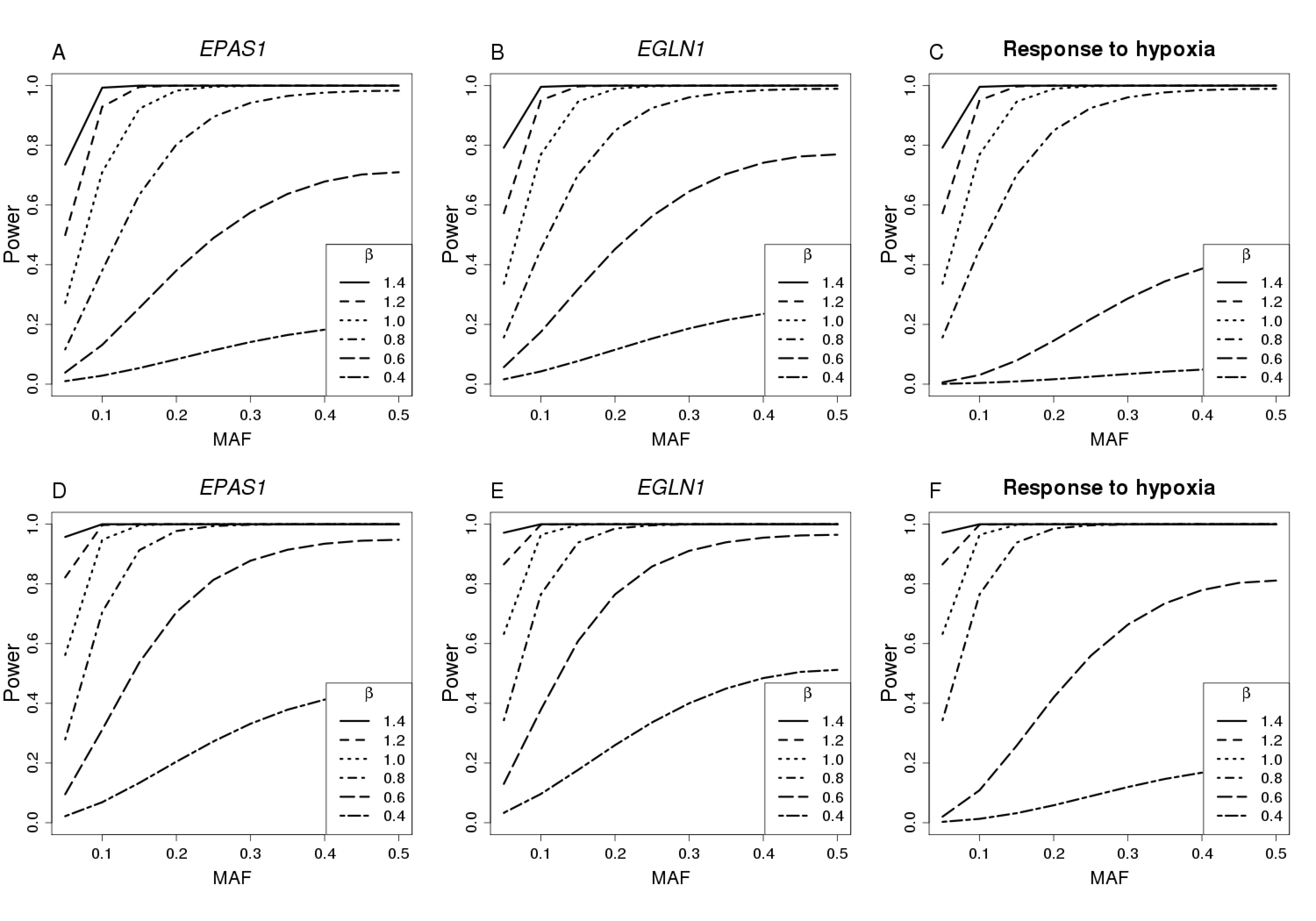

Supplement: Figure S16 — Power plots. The effect of β and MAF on the power of association tests based on the Oromo (A–C) and Amhara (D–F) sample sizes (corrected for the number of SNPs tested within 10 kb from gene) is illustrated for EPAS1 (A and D, 72 SNPs), EGLN1 (B and E, 38 SNPs) and any gene within the Response to Hypoxia gene ontology category (C and F, 1309 SNPs). (TIF) [file pgen.1003110.s016.tif]

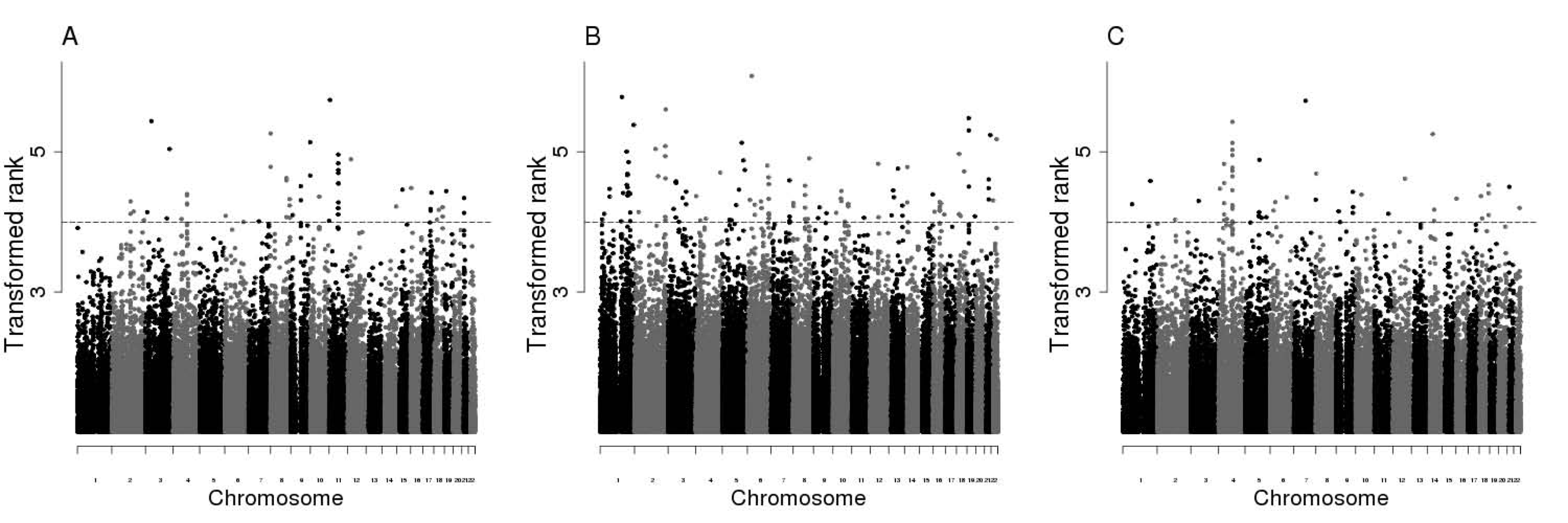

Supplement: Figure S17 — Manhattan plots of PBS and MR transformed rank. Manhattan plots with SNPs with transformed rank less than 0.1 are shown for Amhara PBS (versus Maasai and Luyha) (A), for high altitude Amhara PBS (versus low altitude Amhara and low altitude Oromo) (B), and high altitude Amhara MR (C). The transformed rank is the rank of the SNP in the corresponding distribution divided by the total number of SNPs. The horizontal line represents 0.001 transformed rank. (TIF) [file pgen.1003110.s017.tif]

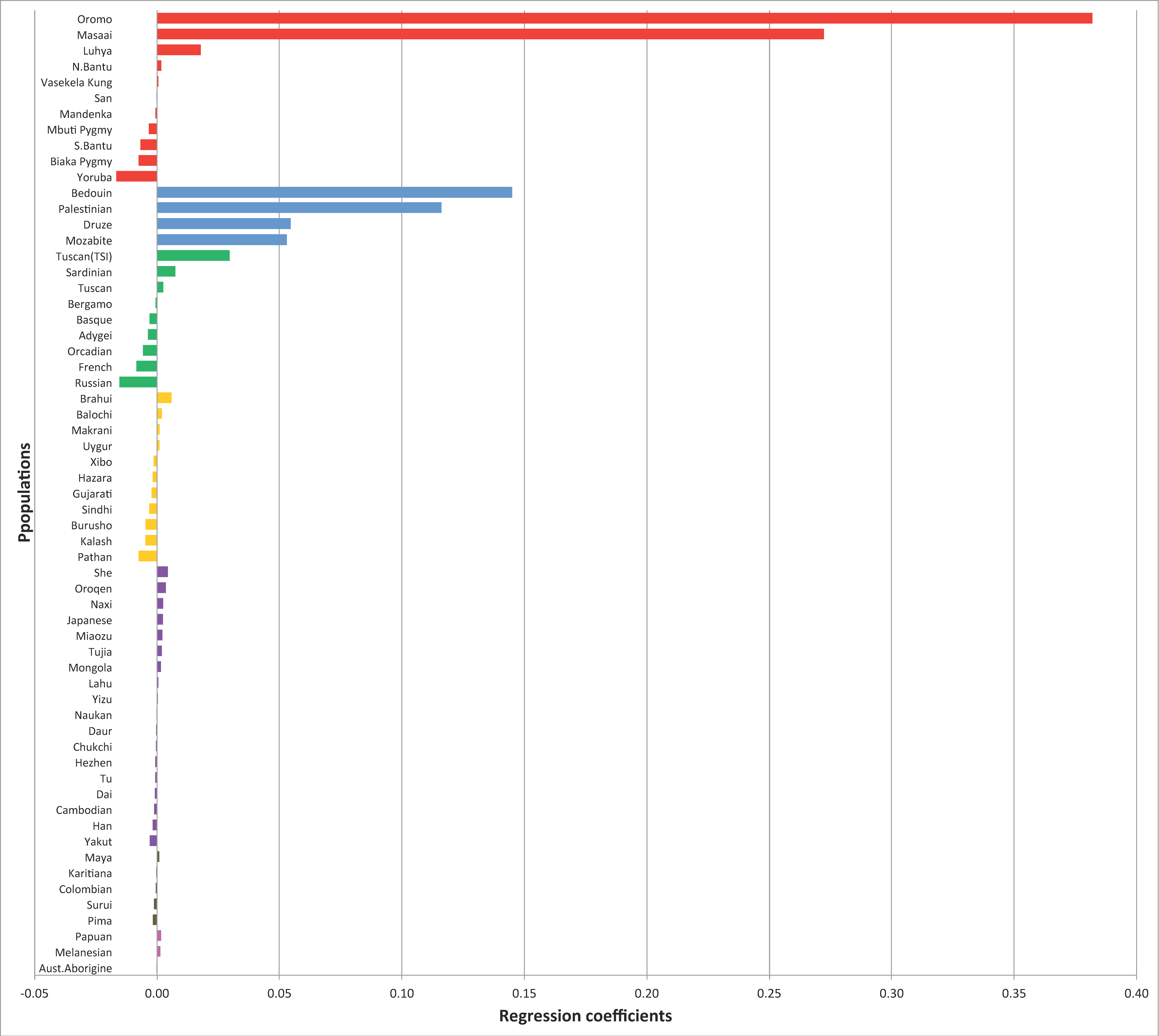

Supplement: Figure S18 — Multiple linear regression coefficients. This plot shows the regression coefficients of each of the 61 populations used to predict the expected allele frequencies in the HA Amhara in the multiple linear regression analysis. Populations have been grouped in Africa, Middle East, Europe, Southwest Asia, East Asia, America and Oceania. In each group, populations have been ordered from larger to smaller coefficients. (TIF) [file pgen.1003110.s018.tif]
