# Supplementary material for: The Genetic Architecture of Adaptations to High Altitude in Ethiopia
Source: PLoS Genet. 2012 Dec 6;8(12):e1003110. doi: 10.1371/journal.pgen.1003110 (PMC3516565; doi:10.1371/journal.pgen.1003110)
Supplement: Table S1 — Sample description for Amhara and Oromo high and low altitude (HA and LA) males and females (mean ± SEM). (PDF) [file pgen.1003110.s021.pdf]

Sample Subset	N	AGE (years)	Height (cm)	Weight (kg)	BMI (kg/m^2)	Pulse (f/min)
HA Amhara males	78	31 + 1	164.8 + 0.8 [**, b]	52.0 + 0.8 [**, b]	19.1 + 0.17 [**, b]	72.7 + 1.5
LA Amhara males	48	34 + 1 [b]	171.1 + 0.9	58.3 + 0.8 [a]	19.9 + 0.24 [b]	75.7 + 1.5
HA Amhara females	24	30 + 2	153.4 + 1.5 [a]	45.2 + 1.5 [**, b]	19.1 + 0.4 [+, c]	84.7 + 3.7
LA Amhara females	12	29 + 3	152.3 + 3.7 [c]	52.8 + 2.3	23.4 + 1.9	84.1 + 3.5
HA Oromo males	35	30 + 1 [**]	170.4 + 1.0	58.9 + 1.0 [*]	20.3 + 0.25 [**]	74.5 + 2.5
LA Oromo males	27	25 + 1	172.2 + 1.1	55.4 + 1.0	18.7 + 0.28	73.6 + 2.2
HA Oromo females	28	27 + 1	157.5 + 1.0 [*]	52.1 + 1.0	21.0 + 0.39	86.8 + 2.9
LA Oromo females	8	26 + 2	161.9 + 1.6	55.7 + 2.9	21.3 + 1.33	86.5 + 2.6

[*] $p < 0.05$ t-test comparing same sex and ethnic group at high and low altitude

[**] $P < 0.01$ t-test comparing same sex and ethnic group at high and low altitude

[+] $0.05 < p < 0.01$ t-test comparing same sex and ethnic group at high and low altitude

[a] $p < 0.05$ t-test comparing Amhara and Oromo of the same sex at one altitude

[b] $P < 0.01$ t-test comparing Amhara and Oromo of the same sex and ethnic group at one altitude

[c] $0.05 < p < 0.01$ t-test comparing Amhara and Oromo of the same sex and ethnic group at one altitude
