# Supplementary material for: The Genetic Architecture of Adaptations to High Altitude in Ethiopia
Source: PLoS Genet. 2012 Dec 6;8(12):e1003110. doi: 10.1371/journal.pgen.1003110 (PMC3516565; doi:10.1371/journal.pgen.1003110)
Supplement: Table S2 — Study phenotypes for Amhara and Oromo high and low altitude (HA and LA) males and females (mean ± SEM). (PDF) [file pgen.1003110.s022.pdf]

Sample Subset	N	Hb (gm/dL)	O_2 Sat (%)	Arterial O_2 content (mlO_2/dL)
HA Amhara males	72	16.8 + 0.13 [*, b]	92.4 + 0.4 [**, b]	21.5 + 0.19
LA Amhara males	41	15.6 + 0.18 [b]	97.2 + 0.2	21.1 + 0.27 [c]
HA Amhara females	21	14.8 + 0.24 [**, b]	93.8 + 0.6 [**, b]	19.4 + 0.32
LA Amhara females	12	13.7 + 0.36 [a, +]	96.9 + 0.4	18.4 + 0.41 [a]
HA Oromo males	35	18.5 + 0.31 [**, +]	86.7+ 0.8 [**]	21.7 + 0.39
LA Oromo males	27	16.7 + 0.22	97.1 + 0.2	21.7 + 0.33
HA Oromo females	27	17.1 + 0.38[**]	84.9 + 0.7 [**]	20.1 + 0.34
LA Oromo females	6	14.8 + 0.34	97.6 + 0.5	20.2 + 0.50

*$p < 0.05$ t-test comparing same sex and ethnic group at high and low altitude

**$P < 0.01$ t-test comparing same sex and ethnic group at high and low altitude

+ $0.05 < p < 0.01$ t-test comparing same sex and ethnic group at high and low altitude

[a] $p < 0.05$ t-test comparing Amhara and Oromo of the same sex at one altitude

[b] $P < 0.01$ t-test comparing Amhara and Oromo of the same sex and ethnic group at one altitude

[c] $0.05 < p < 0.01$ t-test comparing Amhara and Oromo of the same sex and ethnic group at one altitude
