# Supplementary material for: The Genetic Architecture of Adaptations to High Altitude in Ethiopia
Source: PLoS Genet. 2012 Dec 6;8(12):e1003110. doi: 10.1371/journal.pgen.1003110 (PMC3516565; doi:10.1371/journal.pgen.1003110)
Supplement: Table S3 — 20 SNPs with lowest Hb association p-values within Amhara. (PDF) [file pgen.1003110.s023.pdf]

SNP	Chr	N	A1	β	P	Rank	Genes (within 10kb)	Genes (within 100kb)
rs4949254	1	137	G	-0.62	1.09E-05	20		
rs2019842	1	145	G	0.80	3.55E-07	3		
rs6700249	1	145	A	0.74	4.88E-07	4		
rs2184186	1	144	A	0.80	5.16E-07	6		
rs10737884	1	144	G	0.74	7.45E-07	7		
rs1855368	1	146	A	0.74	5.10E-07	5		
rs10803083	1	147	A	0.84	4.96E-08	1		
rs876912	1	143	G	0.78	1.79E-07	2		
rs1378539	2	131	A	0.65	8.02E-06	13		
rs12711718	2	147	A	0.62	8.69E-06	15		
rs4894345	3	146	G	-0.58	9.60E-06	17		*MRPS22,COPB2*
rs16854020	4	140	A	-0.66	2.29E-06	9	*CCDC4*	*SLC30A9*
rs16871658	5	132	A	-0.85	8.56E-06	14		
rs5745616	7	147	A	0.64	1.03E-05	18	*HGF*	
rs2873896	8	133	A	-0.74	1.06E-05	19		
rs2899662	15	147	A	0.91	8.16E-07	8	*RORA*	
rs17204475	15	139	A	0.89	2.65E-06	11	*RORA*	
rs13331158	16	136	A	-0.66	2.42E-06	10		*IRX5*
rs17805042	18	144	A	0.90	9.36E-06	16	*MRO*	*ME2,MAPK4*
rs13051923	21	147	G	0.68	2.72E-06	12		*COL6A2,FTCD,C21orf56,COL6A1*

Only SNPs with MAF <10% and imputation accuracy > 0.9 were tested. In addition to age, sex, BMI (body mass index) and altitude, collection year was also used as covariate since samples were collected ten years apart.
