# Supplementary material for: The Genetic Architecture of Adaptations to High Altitude in Ethiopia
Source: PLoS Genet. 2012 Dec 6;8(12):e1003110. doi: 10.1371/journal.pgen.1003110 (PMC3516565; doi:10.1371/journal.pgen.1003110)
Supplement: Table S4 — 20 SNPs with lowest oxygen saturation association p-values within Amhara. (PDF) [file pgen.1003110.s024.pdf]

SNP	Chr	N	A1	β	P	Rank	Genes (within 10kb)	Genes (within 100kb)
rs10496577	2	149	G	-1.71	1.04E-05	18		
rs17279667	2	161	A	-1.40	1.00E-05	16	*MYO3B*	
rs7659929	4	143	A	-2.13	1.12E-06	3	*ZNF721, PIGG*	*ABCA11*
rs6446322	4	146	G	-1.92	1.64E-07	1	*STK32B*	*CYTL1*
rs10518184	4	161	A	1.32	1.23E-06	4	*FRAS1*	
rs4859905	4	161	A	1.31	2.12E-06	5	*FRAS1*	
rs873455	4	160	A	-1.08	6.97E-06	13	*FRAS1*	
rs157492	5	153	G	-1.43	8.28E-06	14	*GOLPH3*	*MTMR12,PDZD2*
rs11774254	8	154	G	-1.86	4.57E-06	9		
rs17065459	8	161	G	-1.70	1.15E-05	20		
rs10106108	8	161	A	-1.26	1.06E-06	2		
rs11784218	8	159	A	-1.60	3.77E-06	7		
rs4740840	9	151	G	-1.13	4.34E-06	8	*IL33*	*TPD52L3*
rs10904733	10	154	G	-1.26	6.66E-06	12		*PTER,C1QL3*
rs11253992	10	158	A	1.20	5.64E-06	11	*PTER*	*C1QL3*
rs12293066	11	161	A	-1.68	1.02E-05	17		
rs1528635	11	161	G	-1.32	1.05E-05	19		
rs2462165	11	146	A	1.20	4.66E-06	10		
rs416542	14	159	A	1.24	2.74E-06	6	*GALC*	*GPR65*
rs426633	19	161	A	-1.09	8.71E-06	15	*BRUNOL5*	*EDG6,NCLN,GNA15*

Only SNPs with MAF <10% and imputation accuracy > 0.9 were tested. In addition to age, sex, BMI (body mass index) and altitude, collection year was also used as covariate.
