# Supplementary material for: The Genetic Architecture of Adaptations to High Altitude in Ethiopia
Source: PLoS Genet. 2012 Dec 6;8(12):e1003110. doi: 10.1371/journal.pgen.1003110 (PMC3516565; doi:10.1371/journal.pgen.1003110)
Supplement: Table S5 — 20 SNPs with lowest hemoglobin association p-values within high altitude Amhara. (PDF) [file pgen.1003110.s025.pdf]

| SNP | Chr | N | A1 | β | P | Rank | Genes (within 10kb) | Genes (within 100kb) |
|---|---|---|---|---|---|---|---|---|
| rs16854020 | 4 | 92 | A | -0.76 | 2.54E-06 | 1 | *CCDC4* | *SLC30A9* |
| rs13125215 | 4 | 94 | A | -0.66 | 2.63E-05 | 18 | | |
| rs4897037 | 6 | 86 | G | -0.82 | 1.71E-05 | 10 | | *UST* |
| rs9329248 | 8 | 92 | A | 0.86 | 1.61E-05 | 9 | *GATA4,NEIL2* | *FDFT1,CTSB* |
| rs7829680 | 8 | 79 | G | -0.83 | 6.02E-06 | 2 | | |
| rs643273 | 11 | 86 | A | 1.12 | 1.20E-05 | 5 | | *PLAC1L,MS4A2,MS4A3* |
| rs2730985 | 12 | 92 | A | -0.73 | 2.38E-05 | 14 | | |
| rs9315776 | 13 | 94 | A | -0.73 | 2.40E-05 | 16 | *LOC646982* | *LOC646982,FOXO1* |
| rs7494316 | 14 | 94 | A | -0.68 | 2.46E-05 | 17 | *AKAP6* | |
| rs514438 | 15 | 92 | G | -0.85 | 1.47E-05 | 7 | *LCMT2, ADAL* | *TUBGCP4,TP53BP1,ZSCAN29,TGM7,TGM5* |
| rs12911740 | 15 | 89 | A | -0.84 | 2.65E-05 | 19.5 | *TP53BP1* | *HISPPD2A,MAP1A,CKMT1B,TUBGCP4* |
| rs7177146 | 15 | 89 | G | -0.84 | 2.65E-05 | 19.5 | *TP53BP1* | *HISPPD2A,CKMT1B,STRC,MAP1A* |
| rs6493090 | 15 | 92 | A | -0.89 | 1.40E-05 | 6 | *HYPK,SERINC4, SERF2, ELL3* | *hCG_1789710,PDIA3,WDR76,FRMD5, CATSPER2P1,MFAP1,CKMT1A* |
| rs678084 | 15 | 94 | A | -0.86 | 1.99E-05 | 11 | *MFAP1, WDR76* | *FRMD5,CATSPER2P1,SERF2,PDIA3, hCG_1789710,SERINC4,HYPK,ELL3* |
| rs7174241 | 15 | 86 | A | -0.85 | 2.30E-05 | 13 | *WDR76* | *HYPK,CATSPER2P1,SERF2,ELL3,PDIA3, SERINC4,FRMD5,MFAP1,hCG_1789710* |
| rs495880 | 15 | 82 | G | -0.89 | 2.39E-05 | 15 | *WDR76* | *SERF2,ELL3,FRMD5,hCG_1789710, SERINC4,HYPK,MFAP1,PDIA3* |
| rs16962916 | 16 | 80 | A | 0.78 | 1.06E-05 | 4 | | |
| rs2298503 | 18 | 94 | A | 0.82 | 1.48E-05 | 8 | *RAB31* | |
| rs1505267 | 21 | 83 | G | 0.70 | 2.23E-05 | 12 | *CHODL* | |

Only SNPs with MAF <10% and imputation accuracy > 0.9 were tested. In addition to age, sex and BMI (body mass index), collection year was also used as covariate.
