# Supplementary material for: The Genetic Architecture of Adaptations to High Altitude in Ethiopia
Source: PLoS Genet. 2012 Dec 6;8(12):e1003110. doi: 10.1371/journal.pgen.1003110 (PMC3516565; doi:10.1371/journal.pgen.1003110)
Supplement: Table S6 — 20 SNPs with lowest oxygen saturation association p-values within high altitude Amhara. (PDF) [file pgen.1003110.s026.pdf]

SNP	Chr	N	A1	β	P	Rank	Genes (within 10kb)	Genes (within 100kb)
rs17011982	2	98	G	-2.11	5.38E-06	15	*BIRC6*	
rs3731579	2	100	G	-2.15	2.97E-06	6	*BIRC6*	
rs17429032	2	100	G	-2.26	3.62E-06	9.5	*BIRC6*	
rs11683905	2	100	G	-2.26	3.62E-06	9.5	*BIRC6*	
rs12465072	2	98	G	-2.36	4.86E-06	12	*BIRC6*	*TTC27*
rs17321135	2	98	G	-1.89	7.15E-06	20	*CNTNAP5*	
rs7559515	2	99	A	-1.88	7.02E-06	19	*CNTNAP5*	
rs1873393	3	92	G	-3.05	6.95E-06	18	*CHST13, C3orf22*	*UROC1,TR2IT1,ZXDC*
rs7659929	4	92	A	-3.45	1.33E-07	1	*PIGG, ZNF721*	*ABCA11*
rs6446322	4	94	G	-2.38	6.68E-06	16	*STK32B*	*CYTL1*
rs10518184	4	101	A	1.80	4.96E-06	13	*FRAS1*	
rs157492	5	94	G	-2.29	2.81E-06	4	*GOLPH3*	*MTMR12,PDZD2*
rs9403279	6	98	G	1.79	3.21E-06	8		
rs719196	6	88	A	1.92	2.84E-06	5		
rs9376611	6	93	A	1.94	4.56E-06	11		
rs11774254	8	96	G	-2.93	2.46E-06	3		
rs17065459	8	101	G	-2.74	3.10E-06	7		
rs1528635	11	101	G	-2.29	9.28E-07	2		
rs416542	14	99	A	1.80	5.13E-06	14	*GALC*	*GPR65*
rs8081452	17	101	A	1.85	6.78E-06	17		*EFCAB3*
rs876912	1	91	G	0.77	1.04E-05	3		

Only SNPs with MAF <10% and imputation accuracy > 0.9 were tested. In addition to age, sex and BMI (body mass index), collection year was also used as covariate.
