# Supplementary material for: The Genetic Architecture of Adaptations to High Altitude in Ethiopia
Source: PLoS Genet. 2012 Dec 6;8(12):e1003110. doi: 10.1371/journal.pgen.1003110 (PMC3516565; doi:10.1371/journal.pgen.1003110)
Supplement: Table S7 — 20 SNPs with lowest hemoglobin association p-values within low altitude Amhara. (PDF) [file pgen.1003110.s027.pdf]

SNP	Chr	N	A1	β	P	Rank	Genes (within 10kb)	Genes (within 100kb)
rs6657602	1	49	G	2.05	1.03E-05	10	*RYR2*	
rs12105739	2	53	A	-1.73	1.20E-05	13	*ARHGAP15*	
rs7612780	3	52	A	1.61	1.47E-05	18	*OSBPL10*	
rs6550509	3	53	G	-1.12	6.29E-06	3	*ITGA9*	*CTDSPL*
rs12152212	3	53	A	1.46	7.21E-06	6		
rs6813176	4	53	G	1.55	1.47E-06	1	*FLJ46481*	*CRMP1,JAKMIP1*
rs210617	6	52	G	0.99	1.30E-05	16	*DCBLD1, GOPC*	
rs2071825	6	53	A	1.01	7.20E-06	5	*DCBLD1,GOPC*	
rs10457315	6	53	G	0.98	1.32E-05	17	*DCBLD1,GOPC*	
rs10872153	6	52	G	0.93	1.23E-05	14		*DCBLD1, GOPC,NUS1*
rs4946273	6	53	G	0.92	1.80E-05	19		*DCBLD1, GOPC,NUS1*
rs9489238	6	53	A	0.99	7.79E-06	7		*DCBLD1, GOPC,NUS1*
rs1967194	6	53	G	0.92	1.08E-05	11		*DCBLD1, GOPC,NUS1*
rs10087150	8	49	G	1.22	1.24E-05	15		*CPA6*
rs7076094	10	53	G	1.18	4.35E-06	2	*CTNNA3*	
rs1971762	12	52	A	1.08	9.97E-06	9	*ATP5G2*	*ATF7,CALCOCO1*
rs17056224	13	53	A	1.79	8.04E-06	8		
rs10852511	16	42	G	1.05	2.04E-05	20	*PMFBP1*	*HP,HPR,TXNL4B,DHX38*
rs6063285	20	50	G	-1.74	1.10E-05	12		*PREX1*
rs17000823	21	42	G	1.04	6.51E-06	4	*FAM3B*	*MX2,BACE2,MX1*

Only SNPs with MAF <10% and imputation accuracy > 0.9 were tested. Age, sex and BMI (body mass index) were used as covariates.
