# Supplementary material for: The Genetic Architecture of Adaptations to High Altitude in Ethiopia
Source: PLoS Genet. 2012 Dec 6;8(12):e1003110. doi: 10.1371/journal.pgen.1003110 (PMC3516565; doi:10.1371/journal.pgen.1003110)
Supplement: Table S8 — 20 SNPs with lowest oxygen saturation association p-values within low altitude Amhara. (PDF) [file pgen.1003110.s028.pdf]

SNP	Chr	N	A1	β	P	Rank	Genes (within 10kb)	Genes (within 100kb)
rs1020470	3	55	A	-1.04	6.40E-06	14	*SCHIP1,IQCJ*	
rs1873515	4	59	G	-1.48	8.25E-07	2		
rs4735729	8	60	A	-1.35	8.21E-06	18		
rs2490583	9	58	A	-1.52	5.85E-06	12	*RFK*	*GCNT1*
rs2501920	9	60	A	-1.45	5.95E-06	13		*GCNT1,RFK*
rs7847608	9	58	G	-1.50	6.60E-06	15	*GCNT1*	*RFK*
rs6560519	9	60	G	-1.51	4.23E-06	10	*GCNT1*	*RFK*
rs12221280	10	47	G	-1.20	2.28E-06	4	*GPR158*	
rs1352735	11	58	A	-1.44	9.09E-07	3		
rs1982849	12	58	G	-1.50	4.85E-06	11		*PLCZ1,CAPZA3*
rs7980422	12	60	A	-1.18	1.09E-05	20		
rs17116497	15	58	G	-1.64	7.19E-06	16		
rs12323993	15	50	A	-1.56	6.39E-07	1		
rs7172235	15	53	G	-1.12	3.69E-06	8	*PCSK6*	
rs4502305	18	60	A	-1.04	4.11E-06	9		
rs183049	19	59	A	-1.16	2.88E-06	5.5	*SLC6A16*	*FLJ32658,DKKL1,CD37,TEAD2*
rs659555	19	59	A	-1.16	2.88E-06	5.5	*CD37,SLC6A16*	*FLJ32658,TEAD2,DKKL1,PTH2*
rs35270176	19	54	A	-1.36	3.27E-06	7	*LOC199800,CPT1C, PRMT1*	*AP2A1,SCAF1,IRF3,PRR12, RRAS,BCL2L12,TSKS,PRRG2*
rs159095	20	60	A	-1.48	7.29E-06	17		
rs137994	22	59	G	-1.21	8.50E-06	19	*GRAP2*	*TNRC6B,ENTHD1,FAM83F*

Only SNPs with MAF <10% and imputation accuracy > 0.9 were tested. Age, sex and BMI (body mass index) were used as covariates.
