# Supplementary material for: The Genetic Architecture of Adaptations to High Altitude in Ethiopia
Source: PLoS Genet. 2012 Dec 6;8(12):e1003110. doi: 10.1371/journal.pgen.1003110 (PMC3516565; doi:10.1371/journal.pgen.1003110)
Supplement: Table S9 — 20 SNPs with lowest hemoglobin association p-values within Oromo. (PDF) [file pgen.1003110.s029.pdf]

SNP	Chr	N	A1	β	P	Rank	Genes (within 10kb)	Genes (within 100kb)
rs323937	1	94	A	-0.98	1.46E-05	17		
rs6682943	1	94	A	-0.98	1.56E-05	18		
rs1053413	2	94	G	1.31	5.22E-06	7.5	*SLC35F5*	*RABL2A,RPL23AP7*
rs12624301	2	94	G	1.31	5.22E-06	7.5	*SLC35F5*	*RABL2A*
rs4722321	7	91	A	1.08	1.59E-05	19		
rs11765705	7	94	A	1.42	6.77E-06	9	*POU6F2*	
rs834781	7	91	G	-0.99	7.26E-06	10		
rs834787	7	93	G	-1.19	9.61E-06	12		
rs2643268	8	81	G	-1.30	1.60E-05	20		
rs780159	10	94	A	1.30	1.41E-05	15	*ZMIZ1*	*LOC283050*
rs7120319	11	94	A	1.55	3.80E-06	4	*OR51B6,OR51B5*	*HBG1,HBBP1,OR51Q1,OR51I1, HBG2,OR51M1,OR51B2,HBE1,OR51B4*
rs12904003	15	94	G	0.96	2.19E-06	2		*MFGE8,ACAN,HAPLN3*
rs7164649	15	90	G	0.98	3.37E-06	3		*MFGE8,ACAN,HAPLN3*
rs7163586	15	90	G	-0.98	4.42E-06	6		*MFGE8,ACAN,HAPLN3*
rs12443377	15	84	G	-0.97	1.37E-05	14		*MFGE8,ACAN,HAPLN3*
rs12103697	17	94	A	1.39	7.87E-06	11	*SMTNL2*	*GGT6,MYBBP1A,ALOX15,SPNS2,PELP1*
rs894621	17	94	G	1.38	1.81E-06	1	*MSI2*	
rs13306374	18	85	G	0.97	1.23E-05	13	*GALR1*	
rs7276366	21	91	A	1.28	1.45E-05	16		
rs135195	22	75	G	-1.02	3.85E-06	5		

Only SNPs with MAF <10% and imputation accuracy > 0.9 were tested. Age, sex, BMI (body mass index) and altitude were used as covariates.
