# Supplementary material for: The Genetic Architecture of Adaptations to High Altitude in Ethiopia
Source: PLoS Genet. 2012 Dec 6;8(12):e1003110. doi: 10.1371/journal.pgen.1003110 (PMC3516565; doi:10.1371/journal.pgen.1003110)
Supplement: Table S10 — 20 SNPs with lowest oxygen saturation association p-values within Oromo. (PDF) [file pgen.1003110.s030.pdf]

SNP	Chr	N	A1	β	P	Rank	Genes (within 10kb)	Genes (within 100kb)
rs7592938	2	85	G	-3.74	1.25E-05	14	*CTNNA2*	
rs4676890	3	98	A	2.48	8.91E-06	7		
rs164819	5	98	A	-2.25	1.01E-05	10		*TBCA*
rs2569340	5	98	A	-2.81	7.62E-06	6	*PHF15*	*SAR1B*
rs2589403	5	85	A	-2.99	1.23E-05	13	*PHF15*	*SAR1B,SEC24A*
rs7822400	8	98	A	-2.62	5.34E-06	2	*XKR6*	
rs6985930	8	94	G	-2.83	1.11E-05	11	*OXR1*	
rs10109202	8	98	A	-2.54	1.27E-05	18		
rs4876526	8	98	A	-2.54	1.27E-05	18		
rs4876528	8	98	A	-2.54	1.27E-05	18		
rs2123385	8	94	A	-2.54	8.97E-06	8		
rs10106553	8	98	G	-2.54	1.27E-05	18		
rs1452757	8	98	G	-2.78	6.92E-06	4.5		
rs4876314	8	98	A	-2.54	1.27E-05	18		
rs10086147	8	98	A	-2.78	6.92E-06	4.5		
rs902991	10	98	G	2.51	4.36E-06	1	*NEURL*	*SH3PXD2A*
rs3781366	10	95	A	2.32	1.18E-05	12	*NEURL,SH3PXD2A*	
rs12413931	10	98	A	-3.05	6.24E-06	3	*SH3PXD2A*	*NEURL*
rs7102442	11	90	A	2.21	1.01E-05	9		
rs4784651	16	98	A	-2.37	1.26E-05	15	*GNAO1*	*DKFZP434H168,AMFR,LOC283856*

Only SNPs with MAF <10% and imputation accuracy > 0.9 were tested. Age, sex, BMI (body mass index) and altitude were used as covariates.
