# Supplementary material for: The Genetic Architecture of Adaptations to High Altitude in Ethiopia
Source: PLoS Genet. 2012 Dec 6;8(12):e1003110. doi: 10.1371/journal.pgen.1003110 (PMC3516565; doi:10.1371/journal.pgen.1003110)
Supplement: Table S11 — 20 SNPs with lowest hemoglobin association p-values within high altitude Oromo. (PDF) [file pgen.1003110.s031.pdf]

SNP	Chr	N	A1	β	P	Rank	Genes (within 10kb)	Genes (within 100kb)
rs12063638	1	59	A	1.80	1.16E-05	10		*PRDM2,PDPN*
rs323936	1	62	G	-1.23	1.54E-05	14.5		
rs323937	1	62	A	-1.23	1.54E-05	14.5		
rs6682943	1	62	A	-1.24	1.74E-05	16		
rs6532200	4	52	A	1.48	2.25E-05	19	*MMRN1*	*SNCA*
rs12189506	5	58	G	1.55	2.39E-05	20		
rs2108288	7	62	A	1.63	1.52E-05	13	*RAPGEF5*	
rs11765705	7	62	A	1.81	5.87E-06	4	*POU6F2*	
rs721123	7	62	G	1.82	1.11E-06	1	*CNTNAP2*	
rs1997560	8	62	A	1.60	5.91E-06	5		*GDAP1,JPH1*
rs1350172	10	51	G	1.39	1.31E-06	2		*C10orf107*
rs780159	10	62	A	1.63	9.58E-06	9	*ZMIZ1*	*LOC283050*
rs780151	10	60	A	1.60	2.10E-05	18	*ZMIZ1*	
rs7120319	11	62	A	2.06	2.62E-06	3	*OR51B6,OR51B5*	*HBG1,HBBP1,OR51Q1,OR51I1, HBG2,OR51M1,OR51B2,HBE1,OR51B4*
rs11038860	11	57	G	1.40	9.26E-06	7	*CREB3L1*	*MDK,AMBRA1,CHRM4,DGKZ*
rs7476	11	62	A	1.30	1.36E-05	12	*CREB3L1*	*MDK,AMBRA1,CHRM4,DGKZ*
rs10129651	14	51	A	1.36	1.29E-05	11		*SERPINA13,GSC,SERPINA3*
rs3744793	17	62	A	-1.32	9.27E-06	8	*USP36*	*TIMP2,PSCD1*
rs135195	22	48	G	-1.33	8.23E-06	6		
rs138978	22	58	G	-1.29	1.78E-05	17	*SCUBE1*	*TSPO,TTLL12,BIK,MCAT*

Only SNPs with MAF <10% and imputation accuracy > 0.9 were tested. Age, sex and BMI (body mass index) were used as covariates.
