# Supplementary material for: The Genetic Architecture of Adaptations to High Altitude in Ethiopia
Source: PLoS Genet. 2012 Dec 6;8(12):e1003110. doi: 10.1371/journal.pgen.1003110 (PMC3516565; doi:10.1371/journal.pgen.1003110)
Supplement: Table S12 — 20 SNPs with lowest oxygen saturation p-values within high altitude Oromo. (PDF) [file pgen.1003110.s032.pdf]

SNP	Chr	N	A1	β	P	Rank	Genes (within 10kb)	Genes (within 100kb)
rs9557	1	48	G	-4.491	3.73E-06	4	*MAN1A2*	*FAM46C*
rs36235	3	58	A	-3.698	3.97E-06	5	*PRICKLE2*	*PSMD6*
rs7650033	3	58	A	-4.654	2.79E-06	2		*SUCLG2*
rs1173228	5	63	A	-3.468	9.35E-06	11		
rs1392411	5	63	G	-3.987	1.04E-05	12		
rs164819	5	63	A	-3.43	1.30E-05	17		*TBCA*
rs2569340	5	63	A	-3.676	1.17E-05	13	*PHF15*	*SAR1B*
rs4580847	6	55	A	-4.749	2.26E-06	1		*LRFN2*
rs11786531	8	61	G	-4.29	9.30E-06	10		*IMPAD1*
rs9297382	8	59	G	-3.395	8.17E-06	8	*OXR1*	
rs6985930	8	60	G	-4.439	2.87E-06	3	*OXR1*	
rs2123385	8	59	A	-3.535	1.31E-05	18		
rs1452757	8	63	G	-3.647	1.17E-05	14.5		
rs10086147	8	63	A	-3.647	1.17E-05	14.5		
rs7067934	10	63	A	-5.298	5.60E-06	6	*SH2D4B*	*TSPAN14*
rs613587	11	63	G	-4.361	8.90E-06	9	*FLI1*	*KCNJ1*
rs4150282	13	62	A	3.598	1.33E-05	20	*ERCC5*	*C13orf27,LOC121952,BIVM,KDELC1*
rs904864	15	63	G	-4.063	6.55E-06	7		*NOX5,TMEM84,GLCE*
rs1345856	16	48	A	-4.063	1.31E-05	19		
rs6075602	20	63	G	-3.775	1.20E-05	16	*RIN2*	*NAT5,CRNKL1,C20orf26*

Only SNPs with MAF <10% and imputation accuracy > 0.9 were tested. Age, sex and BMI (body mass index) were used as covariates.
