# Supplementary material for: The Genetic Architecture of Adaptations to High Altitude in Ethiopia
Source: PLoS Genet. 2012 Dec 6;8(12):e1003110. doi: 10.1371/journal.pgen.1003110 (PMC3516565; doi:10.1371/journal.pgen.1003110)
Supplement: Table S13 — 20 SNPs with lowest hemoglobin p-values within low altitude Oromo. (PDF) [file pgen.1003110.s033.pdf]

SNP	Chr	N	A1	β	P	Rank	Genes (within 10kb)	Genes (within 100kb)
rs4553221	1	32	A	1.591	2.63E-05	17	*CAMTA1*	
rs696119	1	32	A	-1.395	3.65E-06	5	*C1orf173*	*CRYZ,TYW3*
rs13405506	2	32	A	-1.158	1.19E-06	3	*RAMP1*	*RBM44,UBE2F,LRRFIP1,SCLY*
rs2168494	4	31	A	1.748	1.45E-05	11.5		
rs959628	4	31	A	1.748	1.45E-05	11.5		
rs1378923	4	30	A	1.748	2.14E-05	16		
rs6842580	4	32	G	1.528	3.60E-06	4		
rs17343168	5	32	G	1.806	5.22E-06	6		
rs10484824	6	32	G	1.346	3.88E-07	1	*KIF6*	
rs10456473	6	32	A	1.193	9.44E-07	2	*KIF6*	
rs6458179	6	32	G	0.9942	9.63E-06	8		
rs4548215	8	32	G	1.288	8.68E-06	7		
rs3737147	9	32	G	1.764	1.46E-05	13.5	*NOL8,CENPP*	*OGN,OMD,IARS,SNORA84*
rs7872423	9	32	G	1.764	1.46E-05	13.5	*NOL8,CENPP*	*OGN,OMD,IARS,SNORA84*
rs10999916	10	28	A	1.477	2.64E-05	18	*CDH23*	
rs1327314	13	29	G	1.334	2.80E-05	19		
rs1077918	15	32	A	1.826	1.69E-05	15	*VPS18*	*RHOV,DLL4,CHAC1,INOC1, SPINT1,ZFYVE19,PPP1R14D*
rs1261084	18	31	A	1.074	1.28E-05	9	*TCF4*	
rs5755469	22	25	G	1.377	1.33E-05	10		*ISX*

Only SNPs with MAF <10% and imputation accuracy > 0.9 were tested. Age, sex and BMI (body mass index) were used as covariates.
