# Supplementary material for: The Genetic Architecture of Adaptations to High Altitude in Ethiopia
Source: PLoS Genet. 2012 Dec 6;8(12):e1003110. doi: 10.1371/journal.pgen.1003110 (PMC3516565; doi:10.1371/journal.pgen.1003110)
Supplement: Table S14 — 20 SNPs with lowest oxygen saturation p-values within low altitude Oromo. (PDF) [file pgen.1003110.s034.pdf]

SNP	Chr	N	A1	β	P	Rank	Genes (within 10kb)	Genes (within 100kb)
rs12088462	1	35	A	-1.93	6.56E-06	4	*SLC44A5*	
rs4598448	1	34	A	-1.93	2.02E-05	19	*SLC44A5*	
rs1999493	1	34	G	-1.91	1.10E-05	9	*SLC44A5*	
rs9819197	3	35	A	-1.88	6.60E-06	5		
rs149473	5	35	G	-1.12	4.39E-06	2		
rs12662109	6	35	G	-1.67	5.82E-06	3		
rs17128582	8	34	G	-1.89	9.01E-06	8	*ChGn*	
rs16912021	9	35	A	-1.36	1.26E-05	10	*FANCC*	*C9orf3*
rs12574036	11	33	A	-1.56	1.92E-05	17	*LUZP2*	
rs2512637	11	35	G	-1.65	1.74E-05	15		*ODZ4*
rs10774174	12	35	G	-1.41	1.27E-05	12.5	*PARP11*	*EFCAB4B*
rs12424633	12	35	A	-1.41	1.27E-05	12.5	*PARP11*	*EFCAB4B*
rs7965615	12	35	A	-1.41	1.27E-05	12.5	*PARP11*	*EFCAB4B*
rs11062846	12	35	A	-1.41	1.27E-05	12.5	*PARP11*	*EFCAB4B*
rs3825374	12	34	A	-1.51	1.24E-06	1	*PARP11*	*EFCAB4B*
rs11829730	12	33	G	-1.70	8.90E-06	7	*SOX5*	
rs9520256	13	35	G	1.14	1.95E-05	18		
rs4414463	15	35	A	1.14	8.73E-06	6	*CSPG4*	*SNUPN,ODF3L1,SH3PX3,IMP3*
rs16950701	18	35	A	-1.38	2.14E-05	20	*LIPG*	
rs6038686	20	33	G	-1.96	1.88E-05	16		

Only SNPs with MAF <10% and imputation accuracy > 0.9 were tested. Age, sex and BMI (body mass index) were used as covariates.
