# Supplementary material for: The Genetic Architecture of Adaptations to High Altitude in Ethiopia
Source: PLoS Genet. 2012 Dec 6;8(12):e1003110. doi: 10.1371/journal.pgen.1003110 (PMC3516565; doi:10.1371/journal.pgen.1003110)
Supplement: Table S15 — 20 SNPs with lowest hemoglobin p-values within total Ethiopian sample. (PDF) [file pgen.1003110.s035.pdf]

SNP	Chr	N	A1	β	P	Rank	Genes (within 10kb)	Genes (within 100kb)
rs1574106	1	238	A	-0.52	1.54E-05	15		*TINAGL1,SERINC2,LOC284551*
rs11811630	1	241	A	-0.58	1.66E-05	17	*NCF2*	*APOBEC4,SMG7,RGL1,ARPC5*
rs17453871	2	220	A	0.65	9.72E-06	9		*YWHAQ,ADAM17*
rs2742347	2	223	A	-0.66	1.60E-05	16	*TTN*	*CCDC141*
rs2627037	2	222	A	-0.61	7.30E-06	8	*TTN*	*CCDC141*
rs7590740	2	226	G	-0.70	1.07E-05	11	*TTN*	*CCDC141*
rs6556187	5	241	G	0.85	2.49E-06	4		
rs6978495	7	241	G	0.50	1.72E-05	18	*PDE1C*	
rs1062831	7	230	G	0.70	5.45E-06	7	*RELN*	*SLC26A5*
rs4147310	8	241	G	0.53	1.23E-05	13		
rs7068383	10	201	G	-0.60	1.42E-05	14	*KIAA1217*	*KIAA1217*
rs6480379	10	241	G	0.54	1.01E-05	10		*DDX21,SRGN,VPS26A,KIAA1279*
rs4745975	10	232	G	0.62	1.38E-06	1		*DDX21,SRGN,VPS26A,KIAA1279*
rs1524250	12	226	G	-0.54	1.77E-05	19	*CNOT2*	*KCNMB4*
rs2619133	12	196	A	0.56	1.92E-05	20		*SPIC,MYBPC1*
rs4424941	17	236	A	0.65	3.75E-06	6		*PRKCA,CCDC46,APOH*
rs4305120	17	237	A	0.64	3.60E-06	5		*PRKCA,CCDC46,APOH*
rs8081614	17	241	G	0.66	1.63E-06	2		*PRKCA,CCDC46,APOH*
rs16959046	17	241	G	0.75	2.24E-06	3	*PRKCA*	*APOH*
rs440624	21	226	G	-0.75	1.21E-05	12	*CHODL*	

Only SNPs with MAF <10% and imputation accuracy > 0.9 were tested. Age, sex, BMI (body mass index), collection year, altitude and ethnicity were used as covariates.
