# Supplementary material for: The Genetic Architecture of Adaptations to High Altitude in Ethiopia
Source: PLoS Genet. 2012 Dec 6;8(12):e1003110. doi: 10.1371/journal.pgen.1003110 (PMC3516565; doi:10.1371/journal.pgen.1003110)
Supplement: Table S16 — 20 SNPs with lowest oxygen saturation p-values within total Ethiopian sample. (PDF) [file pgen.1003110.s036.pdf]

SNP	Chr	N	A1	β	P	Rank	Genes (within 10kb)	Genes (within 100kb)
rs12733615	1	259	A	1.24	7.85E-06	7		*UHMK1,UAP1,FLJ13137,SH2D1B*
rs4675041	2	259	A	-1.39	8.70E-06	9		
rs6802224	3	246	G	1.29	3.85E-06	3		
rs7703046	5	253	G	-1.36	8.07E-06	8		
rs11738661	5	256	A	-1.30	1.47E-05	19		
rs337715	5	259	G	-1.43	1.16E-05	15	*KCNN2*	
rs1803989	6	258	A	-1.73	1.38E-05	18	*LOC729603, IGF2R*	*SLC22A2,SLC22A1*
rs1035153	7	259	A	-1.52	9.26E-06	10		
rs6996198	8	259	A	-1.25	9.28E-06	11		*BHLHB5,CYP7B1,LOC401463*
rs10104685	8	255	G	-1.40	1.08E-05	14		
rs2123385	8	252	A	-1.43	1.02E-05	12		
rs1452757	8	259	G	-1.51	1.28E-05	16.5		
rs10086147	8	259	A	-1.51	1.28E-05	16.5		
rs6420192	8	258	A	-1.38	3.90E-06	4		*PARP10,NRBP2,SCRIB,EPPK1, PLEC1,PUF60,GRINA*
rs12380152	9	254	A	-1.57	1.57E-05	20	*PTPRD*	
rs10869434	9	246	A	1.40	2.39E-06	2	*PIP5K1B*	*FAM122A,PIP5K1B*
rs17392931	9	251	G	1.29	4.60E-06	5	*PIP5K1B*	*FAM122A,PIP5K1B*
rs11144066	9	245	G	1.35	2.12E-06	1	*PIP5K1B*	*FAM122A,PIP5K1B*
rs11857947	15	234	A	1.34	5.70E-06	6	*CIB2*	*IDH3A,ACSBG1,TBC1D2B,hCG_38941*
rs176096	17	253	A	1.34	1.04E-05	13	*FLJ45455*	

Only SNPs with MAF <10% and imputation accuracy > 0.9 were tested. Age, sex, BMI (body mass index), collection year, altitude and ethnicity were used as covariates.
