# Supplementary material for: The Genetic Architecture of Adaptations to High Altitude in Ethiopia
Source: PLoS Genet. 2012 Dec 6;8(12):e1003110. doi: 10.1371/journal.pgen.1003110 (PMC3516565; doi:10.1371/journal.pgen.1003110)
Supplement: Table S17 — 20 SNPs with lowest hemoglobin p-values within the total high altitude Ethiopian sample. (PDF) [file pgen.1003110.s037.pdf]

SNP	Chr	N	A1	β	P	Rank	Genes (within 10kb)	Genes (within 100kb)
rs1880418	1	147	A	0.71	1.49E-05	6		
rs7548781	1	154	A	0.87	3.19E-05	17.5	*HSD11B1*	*LAMB3,TRAF3IP3,C1orf107, G0S2,C1orf74,IRF6*
rs13400823	2	140	A	-0.72	2.29E-05	14	*MYT1L*	
rs2624520	5	141	A	0.95	2.08E-05	13		
rs1586656	6	156	G	1.05	2.94E-05	15	*EGFL11*	
rs7801660	7	156	A	0.81	1.92E-05	11	*POU6F2*	
rs6952464	7	155	A	0.79	3.41E-05	19	*POU6F2*	
rs11765705	7	156	A	1.05	4.65E-06	2	*POU6F2*	
rs10951593	7	148	A	1.09	1.01E-05	3	*POU6F2*	
rs10260368	7	156	G	-0.78	1.48E-05	5	*ST7OT2,ST7*	
rs4627213	13	155	A	1.10	3.61E-06	1		
rs2322233	13	150	G	1.05	1.15E-05	4		
rs10129651	14	135	A	0.74	1.83E-05	8		*SERPINA3,GSC,SERPINA13*
rs915378	14	155	A	0.76	2.06E-05	12		
rs4842899	15	144	A	0.81	1.87E-05	10		*KLHL25,AKAP13*
rs7164649	15	145	A	-0.65	3.19E-05	17.5		*ACAN,HAPLN3,MFGE8*
rs7163586	15	149	G	-0.66	3.04E-05	16		*HAPLN3,MFGE8,ACAN*
rs16959046	17	156	G	0.89	1.72E-05	7	*PRKCA*	*APOH*
rs681307	18	155	A	-0.69	1.86E-05	9		

Only SNPs with MAF <10% and imputation accuracy > 0.9 were tested. Age, sex, BMI (body mass index), collection year and ethnicity were used as covariates.
