# Supplementary material for: The Genetic Architecture of Adaptations to High Altitude in Ethiopia
Source: PLoS Genet. 2012 Dec 6;8(12):e1003110. doi: 10.1371/journal.pgen.1003110 (PMC3516565; doi:10.1371/journal.pgen.1003110)
Supplement: Table S18 — 20 SNPs with lowest oxygen saturation p-values within the total high altitude Ethiopian sample. (PDF) [file pgen.1003110.s038.pdf]

SNP	Chr	N	A1	β	P	Rank	Genes (within 10kb)	Genes (within 100kb)
rs6751526	2	164	G	-2.32	1.48E-05	18		
rs7438763	4	162	G	2.08	1.54E-05	20	*LRIT3,RRH*	*EGF,NOLA1,CFI*
rs1990250	4	156	G	2.30	5.97E-06	10	*LRIT3,RRH*	*EGF,NOLA1,CFI*
rs13160737	5	148	G	-2.27	1.18E-05	15	*ZFR*	*MTMR12*
rs9464002	6	162	A	-2.68	2.16E-06	1	*LRRC1*	*C6orf142*
rs6937549	6	164	A	-2.50	1.08E-05	14	*C6orf176*	
rs10269222	7	164	A	-2.15	1.19E-05	16	*SDK1*	
rs10104685	8	160	G	-1.99	4.15E-06	4		
rs2123385	8	158	A	-2.08	2.63E-06	2		
rs1452757	8	164	G	-2.09	5.82E-06	8.5		
rs10086147	8	164	A	-2.09	5.82E-06	8.5		
rs6986020	8	164	G	-1.83	1.24E-05	17		
rs11144066	9	154	G	1.72	1.50E-05	19	*PIP5K1B*	*FAM122A,PIP5K1B*
rs602347	11	141	A	2.09	4.78E-06	5	*EHD1*	*MEN1,ATG2A,MAP4K2,GPHA2, LOC283129,PPP2R5B,CDC42BPG*
rs1647105	12	164	A	1.64	1.07E-05	13		
rs11857947	15	144	A	1.83	5.41E-06	7	*CIB2*	*IDH3A,ACSBG1,TBC1D2B,hCG_38941*
rs10775410	17	164	A	-1.64	7.45E-06	12	*MYO1D*	*CDK5R1,PSMD11*
rs7263002	20	164	G	-2.62	6.28E-06	11	*SNRPB,SNORD119*	*TMC2,ZNF343,TGM6*
rs6114384	20	164	A	-2.63	5.22E-06	6	*SNRPB,SNORD119*	*TMC2,ZNF343,TGM6*
rs8114620	20	159	G	-2.63	2.65E-06	3	*SNRPB,SNORD119*	*TMC2,ZNF343,TGM6*

Only SNPs with MAF <10% and imputation accuracy > 0.9 were tested. Age, sex, BMI (body mass index), collection year and ethnicity were used as covariates.
