# Supplementary material for: The Genetic Architecture of Adaptations to High Altitude in Ethiopia
Source: PLoS Genet. 2012 Dec 6;8(12):e1003110. doi: 10.1371/journal.pgen.1003110 (PMC3516565; doi:10.1371/journal.pgen.1003110)
Supplement: Table S19 — 20 SNPs with lowest hemoglobin p-values within the total low altitude Ethiopian sample. (PDF) [file pgen.1003110.s039.pdf]

SNP	Chr	N	A1	β	P	Rank	Genes (within 10kb)	Genes (within 100kb)
rs12119834	1	85	A	0.98	1.39E-05	11	*PCTK3*	*ELK4,MFSD4,LOC284578,LEMD1*
rs10510829	3	85	G	1.22	1.78E-05	14	*FHIT*	
rs11711620	3	85	G	1.31	1.09E-06	1	*ZBTB20*	
rs1506792	4	82	A	1.06	1.11E-05	9		
rs1506791	4	73	A	1.21	1.01E-05	8		
rs4146409	4	85	A	-0.92	2.54E-06	2		
rs11944671	4	85	A	-0.92	8.30E-06	5		
rs1363073	5	85	A	1.12	1.54E-05	12		
rs13195572	6	85	G	1.16	2.45E-05	19.5		*C6orf138*
rs13198330	6	85	G	1.16	2.45E-05	19.5		*C6orf138*
rs11992396	8	80	A	1.09	1.79E-05	16	*PSD3*	
rs1447293	8	85	A	0.90	8.77E-06	6	*LOC727677*	*POU5F1P1*
rs11142705	9	85	A	0.89	1.25E-05	10	*TRPM3*	
rs12351692	9	77	A	1.35	9.03E-06	7	*CORO2A,TRIM14*	*TBC1D2,NANS*
rs10788462	10	85	A	1.25	1.91E-05	17	*GRID1*	
rs2928011	10	84	A	1.17	1.78E-05	15	*GRID1*	
rs7919376	10	84	A	1.24	2.38E-05	18	*GRID1*	
rs4757615	11	84	A	-0.84	7.40E-06	3	*SAAL1*	*MRGPRX4,TPH1,SERGEF,MRGPRX3,SAA3P*
rs734309	16	84	A	-0.85	1.60E-05	13	*GPT2*	*LOC388272,DNAJA2*
rs6089316	20	80	A	0.82	8.30E-06	4	*TAF4*	*PSMA7,SS18L1,LSM14B*

Only SNPs with MAF <10% and imputation accuracy > 0.9 were tested. Age, sex, BMI (body mass index), collection year and ethnicity were used as covariates.
