# Supplementary material for: The Genetic Architecture of Adaptations to High Altitude in Ethiopia
Source: PLoS Genet. 2012 Dec 6;8(12):e1003110. doi: 10.1371/journal.pgen.1003110 (PMC3516565; doi:10.1371/journal.pgen.1003110)
Supplement: Table S20 — 20 SNPs with lowest oxygen saturation p-values within the total low altitude Ethiopian sample. (PDF) [file pgen.1003110.s040.pdf]

SNP	Chr	N	A1	β	P	Rank	Genes (within 10kb)	Genes (within 100kb)
rs6690390	1	95	A	1.16	8.52E-06	8		
rs11582141	1	95	G	0.97	8.61E-06	9		
rs2792251	1	95	G	0.84	1.42E-05	14	*PBX1*	
rs4551629	1	74	G	-1.20	7.85E-06	7		*LOC441931,ZNF496,ZNF124, VN1R5,FLJ45717*
rs7581685	2	90	G	-1.00	1.82E-05	19		*SPAG16*
rs10458166	6	95	G	-0.93	1.17E-05	11	*PRPF4B*	*C6orf146,C6orf201*
rs980962	6	91	G	-1.15	8.51E-07	2	*ZNF391*	*FKSG83,ZNF184,ZNF204*
rs9373984	6	76	G	0.88	5.88E-06	5	*SCML4*	*SEC63*
rs7784712	7	90	A	-0.98	1.78E-05	17		*HDAC9,TWIST1*
rs2392591	7	90	A	-0.82	6.03E-06	6		*AMPH,LOC340286,VPS41*
rs16890858	8	95	A	-1.04	8.80E-06	10	*SAMD12*	
rs10988467	9	95	A	-0.80	4.83E-06	4	*PRRX2*	*C9orf32,ASB6,C9orf50,PTGES*
rs10786940	10	85	G	0.95	1.89E-05	20		
rs7970151	12	95	A	-1.21	1.78E-05	18		
rs8038032	15	90	G	0.82	1.50E-05	15		*ZNF770,AQR,hCG_1787519*
rs11083340	18	95	A	-0.86	1.61E-05	16		
rs4369752	18	88	A	-0.88	2.16E-06	3		
rs183049	19	92	A	-0.88	1.41E-05	13	*SLC6A16*	*FLJ32658,DKKL1,TEAD2,CD37*
rs659555	19	91	A	-0.90	1.25E-05	12	*SLC6A16,CD37*	*FLJ32658,DKKL1,TEAD2,PTH2*
rs35270176	19	89	A	-1.27	1.73E-07	1	*LOC199800,CPT1C,PRMT1*	*AP2A1,SCAF1,IRF3,PRR12, RRAS,BCL2L12,TSKS,PRRG2*

Only SNPs with MAF <10% and imputation accuracy > 0.9 were tested. Age, sex, BMI (body mass index), collection year and ethnicity were used as covariates.
