# Supplementary material for: The Genetic Architecture of Adaptations to High Altitude in Ethiopia
Source: PLoS Genet. 2012 Dec 6;8(12):e1003110. doi: 10.1371/journal.pgen.1003110 (PMC3516565; doi:10.1371/journal.pgen.1003110)
Supplement: Table S21 — Power calculations within the Ethiopian samples. (PDF) [file pgen.1003110.s041.pdf]

		Tibetans[1]		Amhara			Oromo			Ethiopia		
SNP	Gene	β[2]	half β[3]	N	MAF	Power[3]	N	MAF	Power[3]	N	MAF	Power[3]
rs961154	*EGLN1*	1.70	0.85	141	0.39	100	92	0.41	100	233	0.40	100
rs2790859	*EGLN1*	1.70	0.85	141	0.39	100	92	0.41	100	233	0.40	100
rs1992846	*EPAS1*	0.84	0.42	124	0.36	90	78	0.42	74	202	0.38	99
rs7594278	*EPAS1*	0.52	0.26	131	0.31	49	77	0.24	27	208	0.28	66
rs6544887	*EPAS1*	0.79	0.40	130	0.34	86	86	0.34	70	216	0.34	98
rs17035010	*EPAS1*	0.84	0.42	136	0.39	93	85	0.30	72	221	0.36	99
rs3768729	*EPAS1*	0.80	0.40	133	0.44	91	86	0.44	75	219	0.44	99
rs7583554	*EPAS1*	0.94	0.47	138	0.46	98	90	0.41	89	228	0.44	100
rs7583088	*EPAS1*	0.92	0.46	144	0.30	96	94	0.22	75	238	0.27	100
rs11678465	*EPAS1*	0.85	0.43	144	0.30	92	93	0.22	67	237	0.27	99
rs6712143	*EPAS1*	0.94	0.47	145	0.40	98	92	0.32	86	237	0.37	100
rs4953342	*EPAS1*	0.90	0.45	147	0.21	89	94	0.12	52	241	0.18	97
rs2121266	*EPAS1*	1.02	0.51	147	0.38	99	94	0.30	91	241	0.35	100
rs9973653	*EPAS1*	0.52	0.26	147	0.35	57	94	0.30	37	241	0.33	77
rs1374749	*EPAS1*	0.88	0.44	147	0.47	97	94	0.49	87	241	0.48	100
rs4953353	*EPAS1*	0.97	0.49	147	0.31	98	94	0.31	88	241	0.31	100
rs6756667	*EPAS1*	0.93	0.47	147	0.37	98	94	0.32	86	241	0.35	100
rs7571218	*EPAS1*	0.71	0.36	147	0.50	87	94	0.49	69	241	0.50	98

[1] Genotype-phenotype association beta coefficients for *EGLN1* were obtained from Simonson *et al* [14] while those for *EPAS1* were obtained from Beall *et al* [15].
[2] β indicates the observed linear coefficient for the relationship between SNP genotype and Hb levels.
[3] half β indicates half of the observed linear coefficient for the relationship between SNP genotype and Hb levels.
[3] Power refers to the probability of detecting a significant association ($p<0.05$) between SNP genotype and Hb level given the MAF and the sample size in the Ethiopian populations assuming that the β coefficient is higher than half of the observed in Tibetan.
