# Supplementary material for: The Genetic Architecture of Adaptations to High Altitude in Ethiopia
Source: PLoS Genet. 2012 Dec 6;8(12):e1003110. doi: 10.1371/journal.pgen.1003110 (PMC3516565; doi:10.1371/journal.pgen.1003110)
Supplement: Table S22 — Proportions of Response to Hypoxia genic SNPs relative to the proportion of all other genic SNPs in the tails of the PBS distributions for each population trio tested. * and ** denote support from ≥95% and 99% of bootstrap replicates, respectively. (PDF) [file pgen.1003110.s042.pdf]

	Populations		Hypoxia genes : all other genes PBS tail cut-off		
A	B	C	0.005	0.01	0.05
**Amhara**	Maasai	Luhya	3.40*	3.41**	1.84*
**Amhara**	Maasai	Yoruba	3.432	3.058*	1.929
**Amhara**	LA Oromo	HA Oromo	2.628	1.951	2.203*
**Amhara**	Luhya	Europe	1.899	2.074	1.468
**Amhara**	Luhya	Yoruba	1.151	2.502	1.586
**Amhara**	Maasai	Europe	1.862	2.124	1.406
**HA Amhara**	LA Amhara	LA Oromo	3.31	2.90	1.92*
**HA Amhara**	LA Amhara	Oromo	2.215	2.003	1.557
**HA Amhara**	LA Amhara	Yoruba	1.029	1.843	2.03**
**HA Amhara**	LA Amhara	Maasai	1.675	2.147	1.774
**HA Amhara**	LA Amhara	Europe	2.297	1.619	1.121
**HA Amhara**	LA Amhara	Luhya	0.585	1.167	2.088*
**LA Amhara**	HA Oromo	LA Oromo	1.174	1.528	1.149
**LA Amhara**	HA Amhara	Oromo	0.897	0.81	1.131
**LA Amhara**	HA Amhara	Maasai	1.172	0.995	0.944
**LA Amhara**	HA Amhara	Europe	1.112	0.678	1.026
**LA Amhara**	HA Amhara	Luhya	1.215	0.608	0.939
**LA Amhara**	HA Amhara	Yoruba	1.134	0.567	0.778
**LA Amhara**	HA Amhara	LA Oromo	0.69	0.704	0.895
**Ethiopia**	Maasai	Yoruba	3.991	3.162*	1.578
**Ethiopia**	Luhya	Europe	3.458	2.334	1.226
**Ethiopia**	Luhya	Yoruba	1.358	1.675	1.581
**Ethiopia**	Maasai	Europe	1.184	1.169	1.077
**HA Ethiopia**	LA Ethiopia	Yoruba	2.105	2.367*	1.442*
**HA Ethiopia**	LA Ethiopia	Luhya	0.551	1.378	1.747*
**HA Ethiopia**	LA Ethiopia	Maasai	1.128	0.824	1.323
**HA Ethiopia**	LA Ethiopia	Europe	0.584	0.82	0.971
**LA Ethiopia**	HA Ethiopia	Europe	1.104	1.077	1.247
**LA Ethiopia**	HA Ethiopia	Yoruba	1.756	1.164	0.653
**LA Ethiopia**	HA Ethiopia	Maasai	1.132	0.891	0.9
**LA Ethiopia**	HA Ethiopia	Luhya	1.18	1.176	0.687
**Europe**	Luhya	Oromo	1.74	1.917	1.542
**Europe**	LA Oromo	HA Oromo	2.062	1.463	1.769*
**Europe**	Maasai	Oromo	1.071	2.039	1.737
**Europe**	LA Ethiopia	HA Ethiopia	1.695	1.364	1.332
**Europe**	Luhya	Ethiopia	0.001	1.558	1.411

**Europe**	Luhya	Amhara	0.044	1.149	1.448
**Europe**	LA Amhara	HA Amhara	0.199	1.303	1.213
**Europe**	Maasai	Ethiopia	0.008	1.173	1.375
**Europe**	Maasai	Amhara	0	1.11	1.39
**Luhya**	LA Amhara	HA Amhara	2.274	1.675	1.55
**Luhya**	LA Ethiopia	HA Ethiopia	2.317	1.686	1.357
**Luhya**	Ethiopia	Europe	1.202	1.923	1.741
**Luhya**	Amhara	Europe	1.174	1.693	1.818*
**Luhya**	Oromo	Europe	1.722	1.431	1.286
**Luhya**	Amhara	Yoruba	1.667	2.068	1.494
**Luhya**	LA Oromo	HA Oromo	1.952	1.513	1.222
**Luhya**	Ethiopia	Yoruba	1.13	1.4	1.363
**Luhya**	Oromo	Yoruba	0.881	1.388	1.38
**Luhya**	Maasai	Amhara	0.457	1.151	0.953
**Luhya**	Maasai	Oromo	0.516	0.584	0.888
**Maasai**	Amhara	Europe	3.022	2.468	1.704
**Maasai**	LA Amhara	HA Amhara	4.125	3.497*	1.706
**Maasai**	Ethiopia	Europe	1.851	2.408	1.59
**Maasai**	LA Ethiopia	HA Ethiopia	2.343	2.645	1.542
**Maasai**	LA Oromo	HA Oromo	4.41	2.683	0.964
**Maasai**	Oromo	Europe	1.541	1.408	1.377
**Maasai**	Amhara	Yoruba	1.675	1.122	1.202
**Maasai**	Ethiopia	Yoruba	1.746	0.873	1.048
**Maasai**	Amhara	Luhya	1.753	1.144	0.821
**Maasai**	Oromo	Yoruba	1.689	1.127	0.824
**Maasai**	Oromo	Luhya	1.194	0.785	0.972
**Oromo**	LA Amhara	HA Amhara	2.69	2.19	2.114**
**Oromo**	Maasai	Luhya	2.988	1.756	1.445
**Oromo**	Maasai	Yoruba	3.464	2.002	1.038
**Oromo**	Luhya	Europe	1.261	1.719	1.165
**Oromo**	Luhya	Yoruba	0.916	1.391	1.116
**Oromo**	Maasai	Europe	1.175	1.261	0.936
**HA Oromo**	LA Amhara	LA Oromo	1.858	1.567	1.155
**HA Oromo**	LA Oromo	Amhara	1.324	1.857	0.835
**HA Oromo**	LA Oromo	Europe	0.502	1.622	1.161
**HA Oromo**	LA Oromo	Yoruba	0.001	1.353	1.089
**HA Oromo**	LA Oromo	Maasai	0.606	0.913	0.993
**HA Oromo**	LA Oromo	Luhya	0.53	0.266	1.159
**LA Oromo**	HA Oromo	Europe	2.869	1.972	1.355
**LA Oromo**	HA Oromo	Yoruba	3.754	1.903	1.162

**LA Oromo**	HA Oromo	Amhara	1.999	1.558	1.166
**LA Oromo**	HA Oromo	Maasai	1.671	1.478	1.064
**LA Oromo**	LA Amhara	HA Amhara	0.219	1.189	1.393
**LA Oromo**	LA Amhara	HA Oromo	0.889	0.898	0.837
**LA Oromo**	HA Oromo	Luhya	0.53	0.266	1.159
**Yoruba**	LA Ethiopia	HA Ethiopia	1.588	1.309	1.587
**Yoruba**	LA Amhara	HA Amhara	1.057	2.15	1.617
**Yoruba**	LA Oromo	HA Oromo	2	1.259	1.32
**Yoruba**	Maasai	Amhara	1.71	1.092	1.312
**Yoruba**	French	Oromo	0.532	1.703	1.808
**Yoruba**	Maasai	Ethiopia	1.597	1.077	1.227
**Yoruba**	Maasai	Oromo	1.597	0.799	1.213
**Yoruba**	Luhya	Ethiopia	2.149	1.074	0.773
**Yoruba**	Luhya	Oromo	2.2	1.1	0.737
**Yoruba**	Luhya	Amhara	2.181	1.091	0.739
