# Supplementary material for: The Genetic Architecture of Adaptations to High Altitude in Ethiopia
Source: PLoS Genet. 2012 Dec 6;8(12):e1003110. doi: 10.1371/journal.pgen.1003110 (PMC3516565; doi:10.1371/journal.pgen.1003110)
Supplement: Table S23 — List of the 20 SNPs with the largest Amhara PBS (versus Maasai and Luya). (PDF) [file pgen.1003110.s043.pdf]

SNP	Chr	Nt. pos.	Rank	Gene (within 10kb)	Gene (within 100kb)
rs619660	3	43002953	2	LOC729085	*C3orf39,ZNF662,CCBP2*
rs9853065	3	174475785	5	NA	*NA*
rs6994475	8	1260833	3	NA	*NA*
rs6558447	8	1261463	9	NA	*NA*
rs2357735	8	115943237	13	NA	*NA*
rs10112994	8	116016470	14	NA	*NA*
rs1888076	9	72945254	17	TRPM3	*TRPM3*
rs7860423	9	140075368	4	CACNA1B	*NA*
rs11137360	9	140077378	12	CACNA1B	*NA*
rs7940567	11	7078885	1	RBMXL2	*ZNF214,NLRP14*
rs4375446	11	66667402	10	FBXL11	*RHOD,SYT12*
rs674499	11	66720001	11	FBXL11	*ANKRD13D,ADRBK1*
rs4542419	11	66727173	6	FBXL11	*ANKRD13D,ADRBK1*
rs3927807	11	66755431	8	FBXL11	*SSH3,ADRBK1,ANKRD13D*
rs1638566	11	66880909	15.5	POLD4,CLCF1	*PTPRCAP,CORO1B,GPR152,TBC1D10C,KIAA1394,RPS6KB2 CABP4,PPP1CA,ADRBK1,ANKRD13D,SSH3,RAD9A,FBXL11*
rs1638567	11	66881799	15.5	POLD4,CLCF1	*PTPRCAP,CORO1B,GPR152,TBC1D10C,KIAA1394,RPS6KB2 CABP4,PPP1CA,ADRBK1,ANKRD13D,SSH3,RAD9A,FBXL11*
rs10842162	12	23444386	7	NA	*NA*
rs735480	15	42939663	19	NA	*TRIM69,C15orf43*
rs1011489	16	5607394	18	NA	*NA*
rs2306192	19	14004016	20	IL27RA,RLN3	*LOC113230,PRKACA,ASF1B,SAMD1,PODNL1,LOC90379,RFX1*

Only SNPs with imputation accuracy > 0.9 were tested.
