# Supplementary material for: The Genetic Architecture of Adaptations to High Altitude in Ethiopia
Source: PLoS Genet. 2012 Dec 6;8(12):e1003110. doi: 10.1371/journal.pgen.1003110 (PMC3516565; doi:10.1371/journal.pgen.1003110)
Supplement: Table S24 — 20 SNPs with largest high altitude Amhara PBS (versus low altitude Amhara and low altitude Oromo). (PDF) [file pgen.1003110.s044.pdf]

SNP	Chr	Nt. pos.	Rank	Genes (within 10kb)	Genes (within 100kb)
rs2814778	1	157441307	2	*DARC,CADM3*	*FCER1A*
rs1487529	1	192399145	12		
rs10863766	1	204892228	17	*DYRK3*	*LGTN,RASSF5,MAPKAPK2*
rs7539843	1	242957549	5		*FAM152A,C1orf101*
rs10180234	2	153095625	11	*FMNL2*	
rs349003	2	222660565	10		
rs349010	2	222663592	14		
rs3768889	2	225073300	3	*CUL3*	*FAM124B*
rs13181143	5	140186928	9	*PCDHA1, PCDHA2, PCDHA3, PCDHA4, PCDHA5, PCDHA6, PCDHA7*	*PCDHAC1,PCDHA8,PCDHA9, PCDHA10,PCDHA11, PCDHA12,PCDHA13*
rs4377754	5	153096868	16	*GRIA1*	
rs7764351	6	32529609	1	*HLA-DRA*	*HLA-DRB5,BTNL2,HLA-DRB1,C6orf10,HLA-DRB6*
rs7753021	6	145310595	19		*UTRN*
rs10094452	8	117810022	15	*EIF3H*	*C8orf53*
rs2048803	12	57841104	18		
rs1983521	14	21688320	20		
rs16940947	18	20533441	13		
rs10416242	19	13611618	4		*CCDC130*
rs1422837	19	13615667	6		*CCDC130*
rs398717	21	42874233	7	*SLC37A1*	*PDE9A,SLC37A1,RSPH1*
rs133352	22	40763968	8	*WBP2NL*	*NDUFA6,CYP2D6,NAGA,FAM109B, C22orf32,LOC339674,CENPM,SEPT3*

Only SNPs with imputation accuracy > 0.9 were tested.
