# Supplementary material for: The Genetic Architecture of Adaptations to High Altitude in Ethiopia
Source: PLoS Genet. 2012 Dec 6;8(12):e1003110. doi: 10.1371/journal.pgen.1003110 (PMC3516565; doi:10.1371/journal.pgen.1003110)
Supplement: Table S25 — SNPs with oxygen saturation (O2 Sat) association p-value<0.05 among the top 20 Amhara PBS SNPs. (PDF) [file pgen.1003110.s045.pdf]

Test1	Test1	SNP	Chr	Nt. pos.	Hb P	O_2 Sat P	PBS P	Genes (within 10kb)	Genes (within 100kb)
PBS	O_2 Sat	rs619660	3	43002953	7.00E-02	1.70E-02	3.60E-06	*LOC72985*	*C3orf39,ZNF662,CCBP2*
PBS	O_2 Sat	rs9853065	3	1.74E+08	3.80E-01	2.70E-02	9.10E-06		
