# Supplementary material for: The Genetic Architecture of Adaptations to High Altitude in Ethiopia
Source: PLoS Genet. 2012 Dec 6;8(12):e1003110. doi: 10.1371/journal.pgen.1003110 (PMC3516565; doi:10.1371/journal.pgen.1003110)
Supplement: Table S26 — 20 SNPs with largest high altitude Amhara PBS or MR and Hb or O2 sat association pvalue<0.05 within HA Amhara. (PDF) [file pgen.1003110.s046.pdf]

Test 1	Test 2	SNP	Chr	Nt. pos.	Hb P	O_2 Sat P	Genes (within 10kb)	Genes (within 100kb)
PBS	O_2 Sat	rs7753021	6	145310595	9.50E-01	5.40E-03		*UTRN*
PBS	O_2 Sat	rs1983521	14	21688320	6.70E-01	3.50E-02		
PBS	Hb	rs16940947	18	20533441	2.70E-02	5.30E-01		
MR	Hb	rs926130	21	15136788	8.66E-03	5.67E-01		
