# Supplementary material for: The Genetic Architecture of Adaptations to High Altitude in Ethiopia
Source: PLoS Genet. 2012 Dec 6;8(12):e1003110. doi: 10.1371/journal.pgen.1003110 (PMC3516565; doi:10.1371/journal.pgen.1003110)
Supplement: Table S27 — 20 SNPs with largest high altitude Amhara MR score. (PDF) [file pgen.1003110.s047.pdf]

SNP	Chr	Nt. pos.	Rank	Genes (within 10kb)	Genes (within 100kb)
rs682600	1	199730648	14	*CSRP1*	*PHLDA3,LOC376693,TNNI1,LAD1*
rs6810492	4	11833486	18		
rs2660343	4	41672523	15	*WDR21B*	*TMEM33,SLC30A9*
rs2660342	4	41672561	8	*WDR21B*	*TMEM33,SLC30A9*
rs12510722	4	100366124	4	*ADH6*	*ADH1A,ADH1B,ADH4*
rs2173199	4	100390402	5		*ADH1A,ADH1B,ADH1C,ADH6*
rs6532814	4	100392991	6		*ADH1A,ADH1B,ADH1C,ADH6*
rs1826909	4	100436766	19	*ADH1A,ADH1B*	*ADH1C,ADH6*
rs13103321	4	100439263	12	*ADH1A,ADH1B*	*ADH1C,ADH6*
rs1353621	4	100460598	9	*ADH1B*	*ADH1C,ADH1A,ADH7*
rs7661978	4	100503222	10		*ADH1B,ADH1C,ADH7,ADH1A*
rs729147	4	100552290	2	*ADH7*	*ADH1C,C4orf17,ADH1B*
rs325502	5	104036032	7		
rs2662891	7	85327629	1		
rs1994056	8	3610422	11	*CSMD1*	
rs1412060	9	121329170	20		
rs4842631	12	87450918	13	*KITLG*	
rs9323043	14	40618377	3		
rs8091228	18	69134448	16		
rs926130	21	15136788	17		

Only SNPs with imputation accuracy > 0.9 were tested.
