# Supplementary material for: The Genetic Architecture of Adaptations to High Altitude in Ethiopia
Source: PLoS Genet. 2012 Dec 6;8(12):e1003110. doi: 10.1371/journal.pgen.1003110 (PMC3516565; doi:10.1371/journal.pgen.1003110)
Supplement: Table S28 — 40 CpG sites with highest high versus low methylation difference within Oromo. TSS denotes transcription start site. (PDF) [file pgen.1003110.s048.pdf]

CpG	Chr	Nt. pos.	P	Rank	Distance to TSS	Genes
cg04286933	22	37802689	2.69E-07	1	393	*APOBEC3G*
cg22198623	16	55259369	7.18E-07	2	109	*MT1G*
cg14056644	4	111778554	1.27E-06	3	597	*PITX2*
cg09931793	9	113130082	1.54E-06	4	452	*OR2K2*
cg15089487	15	68972306	1.95E-06	5	499	*THAP10*
cg14785479	22	19122535	6.52E-06	6	389	*SCARF2*
cg20988728	22	30018502	8.99E-06	7	28	*MGC17330*
cg07200897	3	123995511	9.24E-06	8	171	*HSPBAP1*
cg07426848	1	151788336	9.63E-06	9	22	*S100A3*
cg11237738	4	5578196	1.06E-05	10	412	*C4orf6*
cg24237576	16	2225776	1.08E-05	11	693	*DNASE1L2*
cg13006591	4	38508060	1.20E-05	12	505	*TLR6*
cg21053015	22	38076000	1.25E-05	13	100	*SYNGR1*
cg03533858	1	2314256	1.46E-05	14	1403	*MORN1*
cg02592124	2	202811724	1.96E-05	15	157	*SUMO1*
cg10313633	11	44929466	2.55E-05	16	456	*TP53I11*
cg10574499	16	66476255	2.65E-05	17	27	*UNQ2446*
cg18410627	9	128717703	2.88E-05	18	829	*RALGPS1*
cg15983520	8	145699914	2.93E-05	19	317	*GPT*
cg26856388	7	64975330	3.11E-05	20	362	*VKORC1L1*
cg13210534	11	111289538	3.52E-05	21	829	*HSPB2*
cg08611714	17	36877384	3.55E-05	22	220	*KRTHA2*
cg04081402	22	29333587	4.07E-05	23	426	*TCN2*
cg24585690	5	135259413	4.10E-05	24	2	*IL9*
cg17043155	2	75727427	4.15E-05	25	7	*MRPL19*
cg22036988	3	142253035	4.33E-05	26	91	*SPSB4*
cg10525372	6	112482026	4.40E-05	27	55	*WISP3*
cg19464252	16	30582734	4.43E-05	28	1155	*FBS1*
cg21830413	10	70554257	4.47E-05	29	295	*VPS26*
cg05670596	3	46423500	4.84E-05	30	225	*CCRL2*
cg00795812	2	242450682	5.78E-05	31	951	*PDCD1*
cg18168989	9	76834070	5.90E-05	32	940	*C9orf41*
cg17964955	10	48010942	5.93E-05	33	55	*RBP3*
cg10150813	4	25474276	5.94E-05	34	734	*KIAA0746*
cg08859675	19	10424771	6.09E-05	35	134	*PDE4A*
cg22396755	1	21867684	6.16E-05	36	702	*RAP1GA1*
cg22858308	6	143137306	6.69E-05	37	369	*HIVEP2*
cg06852744	7	44109498	6.73E-05	38	987	*AEBP1*
cg13439730	16	31054500	7.23E-05	39	152	*PRSS8*
cg15901783	13	76359427	7.51E-05	40	901	*KCTD12*
