# Supplementary material for: The Genetic Architecture of Adaptations to High Altitude in Ethiopia
Source: PLoS Genet. 2012 Dec 6;8(12):e1003110. doi: 10.1371/journal.pgen.1003110 (PMC3516565; doi:10.1371/journal.pgen.1003110)
Supplement: Table S29 — 40 CpG sites with highest high versus low methylation difference within Amhara. TSS denotes transcription start site. (PDF) [file pgen.1003110.s049.pdf]

CpG	Chr	Nt. pos.	P	Rank	Distance to TSS	Genes
cg16351002	10	124600091	8.13E-06	1	208	*CUZD1*
cg11378686	6	43444256	4.32E-05	2	903	*ZNF318*
cg05244766	11	67107075	8.03E-05	3	787	*GSTP1*
cg22445920	5	150663371	1.38E-04	4	149	*SLC36A3*
cg12026956	5	72147764	2.03E-04	5	490	*TNPO1*
cg08385610	8	20156963	2.23E-04	6	120	*LZTS1*
cg10453758	3	133862880	2.50E-04	7	1264	*ACAD11*
cg19764399	13	47567117	2.81E-04	8	124	*MED4*
cg00922727	6	109521816	3.18E-04	9	154	*SESN1*
cg23364287	3	48729780	3.99E-04	10	70	*IHPK2*
cg04609640	1	45026679	4.21E-04	11	715	*VMD2L2*
cg23743114	17	31352509	4.83E-04	12	616	*CCL15*
cg27626899	15	89276929	5.27E-04	13	149	*HDDC3*
cg23323879	15	63291587	5.85E-04	14	723	*CILP*
cg16746462	5	96296194	6.36E-04	15	908	*LNPEP*
cg07246225	2	144994129	6.50E-04	16	257	*ZFHX1B*
cg05449607	6	111386305	6.78E-04	17	151	*C6orf51*
cg10167296	1	204747251	7.16E-04	18	251	*RASSF5*
cg10051054	1	3659032	7.66E-04	19	210	*CCDC27*
cg23487586	16	83873424	8.29E-04	20	641	*MGC22001*
cg09450020	7	89679371	8.31E-04	21	435	*STEAP2*
cg21850254	19	42651693	8.43E-04	22	129	*ZNF570*
cg08866753	1	39814648	8.66E-04	23	355	*PABPC4*
cg00600684	10	122600148	8.71E-04	24	537	*BRWD2*
cg05028306	4	17187761	8.95E-04	25	264	*LAP3*
cg09180926	15	89338658	9.11E-04	26	150	*PRC1*
cg04868764	15	38924311	9.17E-04	27	773	*SPINT1*
cg13726191	4	15549131	9.20E-04	28	62	*FGFBP1*
cg08303146	11	2423231	9.71E-04	29	NA	*KCNQ1*
cg18493238	10	37454267	1.00E-03	30	524	*ANKRD30A*
cg02449978	3	48205171	1.01E-03	31	366	*CDC25A*
cg03077492	5	43448852	1.02E-03	32	623	*CCL28*
cg24134767	11	113350848	1.03E-03	33	272	*HTR3A*
cg23863670	5	43447686	1.04E-03	34	543	*CCL28*
cg12820481	1	209915383	1.05E-03	35	207	*NEK2*
cg07123548	19	45588003	1.09E-03	36	69	*HIPK4*
cg16791508	12	51001958	1.13E-03	37	520	*KRTHB3*
cg09126273	12	15367034	1.17E-03	38	NA	*PTPRO*
cg07710481	13	87123385	1.21E-03	39	514	*SLITRK5*
cg16961218	18	74930131	1.24E-03	40	254	*ATP9B*
