# Supplementary material for: The Genetic Architecture of Adaptations to High Altitude in Ethiopia
Source: PLoS Genet. 2012 Dec 6;8(12):e1003110. doi: 10.1371/journal.pgen.1003110 (PMC3516565; doi:10.1371/journal.pgen.1003110)
Supplement: Text S1 — Sampled populations and their ecology. (DOCX) [file pgen.1003110.s050.docx]

**Text S1. Sampled populations and their ecology**

The Simien Plateau in Northwest Ethiopia is cut by streams and rivers and has cliffs and land areas sloping down to canyons. The high altitude (HA) Amhara are agropastoralists living in a temperate Afro-alpine ecosystem in the Simien Mountains National Park at altitudes ranging from 3500-4100 meters (m). Altitudes above 2500m on the East African Plateau have been inhabited for at least 5 thousand years (ky) and altitudes around 2300-2400m for more than 70ky [[1](#_ENREF_1),[2](#_ENREF_2)]. Linking historical and modern ethnic groups in Ethiopia with prehistoric sites is not feasible with current knowledge. This paper considers that 5ky is a reasonable lower estimate for human habitation of this area. The Bale Plateau in Southeast Ethiopia is a flat mesa with sharp relief down to the lowlands. The HA Oromo are pastoralists herding cattle, sheep and goats and living in a temperate Afro-alpine ecosystem in the Bale Mountains National park and reside on the Sanetti Plateau at 4000-4100m. The HA areas of the Bale Plateau have been inhabited by Oromo since the early 1500s according to historical records [[3](#_ENREF_3),[4](#_ENREF_4)]. The two plateaus are about 500 miles apart and the intervening terrain is not a continuous plateau as the Tibetan or Andean, instead it is a mosaic of terrain generally above 1500m and punctuated by lowlands including the Great Rift Valley. The Amhara language is sub-classified as Ethio-Semitic and Oromo as Eastern Cushitic in the Afroasiatic language group [[5](#_ENREF_5)] **(**[http://www.ethnologue.com**/**](http://www.ethnologue.com/) **- accessed February 27, 2012).**

Data were collected in field laboratories in the four communities. The Amhara samples provided data during December 1995, April 2005, December 2005 and January 2006. In 2005-6, they were studied at a field laboratory established in a park guard camp at 3700m. In 1995, they were studied at an altitude of 3530m [[6](#_ENREF_6),[7](#_ENREF_7)]. Ambient conditions at the time of morning calibrations in 2005 and 2006 averaged 498 mmHg, 6 °C and 36% relative humidity at the HA site, 659 mmHg, 22 °C, and 36% relative humidity at the LA site. The Oromo samples provided data during December 2005 and January 2006 when ambient conditions averaged 469mm Hg and 3°C at the HA site and 635 mmHg, 15°C at the LA site. They were studied at a field laboratory established at the Wolf Research Camp at 4000m and in Melkibuta town at 1500m.

The Amhara reported occupations as farmers and, at LA, farmers and traders. The HA sample reported eating a diet based on barley injera (a flat crepe-like bread made of fermented dough) with sauce (wot) of potato or beans. The LA Amhara community was exposed to malaria and schistosomiasis and had a high prevalence of visible goiter and iron deficiency. Such individuals were excluded from the sample. The HA Oromo sample reported eating a diet of teff injera with potato or bean wot and some meat and dairy products. The LA Oromo were farmers, traders, and government officials such as teachers; they were also exposed to malaria.

REFERENCES

1. Aldenderfer MS (2003) Moving Up in the World; Archaeologists seek to understand how and when people came to occupy the Andean and Tibetan plateaus. American Scientist 91: 542-549.

2. Pleurdeau D (2006) Human Technical Behavior in the African Middle Stone Age: The Lithic Assemblange of Porc-Epic Cave (Dire Dawa, Ethiopia). African Archaeological Review 22: 177-197.

3. Hassen M (1990) The Oromo of Ethiopia: a history, 1570-1860. Great Britain: Cambridge University Press.

4. Lewis HS (1966) The Origins of the Galla and Somali. The Journal of African History 7: 27-46.

5. Lewis MP, editor (2009) Ethnologue: Languages of the World, Sixteenth edition. Dallas, Tex.: SIL International.

6. Beall CM, Decker MJ, Brittenham GM, Kushner I, Gebremedhin A, et al. (2002) An Ethiopian pattern of human adaptation to high-altitude hypoxia. Proc Natl Acad Sci U S A 99: 17215-17218.

7. Beall CM, Gebremedhin A, Brittenham GM, Shamebo M (1997) Blood pressure variation among Ethiopians at 3530m on the Simien Plateau. Annals of Human Biology 24: 333-342.
