# Supplementary material for: The Genetic Architecture of Adaptations to High Altitude in Ethiopia
Source: PLoS Genet. 2012 Dec 6;8(12):e1003110. doi: 10.1371/journal.pgen.1003110 (PMC3516565; doi:10.1371/journal.pgen.1003110)
Supplement: Text S2 — The genetic structure of the Amhara and Oromo populations. (DOCX) [file pgen.1003110.s051.docx]

**Text S2. The genetic structure of the Amhara and Oromo populations**

In order to investigate the genetic relationship between Amhara and Oromo and other populations, we used genotype data in the two Ethiopian populations for SNPs that had previously been typed also in a set of 60 worldwide populations, namely 52 Human Genome Diversity Project (HGDP) populations, 4 HapMap populations (Gujarati Indians in Houston, Texas (GIH); Maasai in Kinyawa, Kenya (MKK); Luhya in Webuye, Kenya (LWK); Toscani in Italia (TSI)), and 4 populations from Hancock et al [[1](#_ENREF_1)]. A neighbor-joining tree shows the relationships of the Ethiopian populations to the worldwide populations (Figure S1). As expected, Oromo and Amhara cluster closely together in the tree and occupy an intermediate position between African (with the exception of the Mozabites) and non-African populations. This position is further supported by the Bayesian clustering analysis performed using the program STRUCTURE [[2](#_ENREF_2)]. In this analysis, 3 different sets of 57652 SNPs were used to infer the ancestral composition of each population assuming 7 ancestral groups. The STRUCTURE plots clearly show that Ethiopian populations share ancestral components with sub-Saharan African and Middle Eastern populations falling in the middle of the ancestry gradient between these two groups of populations (Figure S2. We also calculated the haplotype diversity and compared it to that observed in the worldwide populations. Interestingly, the Oromo (0.822) and Amhara (0.810) haplotype diversity values are as high as or higher than the highest values [[3](#_ENREF_3)] observed in the HGDP, i.e. Bantu (0.818), Biaka Pygmies (0.815), Yoruba (0.815) and Mandenka (0.807); this is true regardless of altitude (0.798 for HA Amhara; 0.803 for LA Amhara, 0.813 for HA Oromo, and 0.813 for LA Oromo).

To look more closely at the relationship between the Amhara and the Oromo, we performed principal component analysis (PCA) using the genotype data from 13,000 random autosomal SNPs for all Ethiopian individuals. In this analysis, PC1, which explains 0.78% of the variance, separates the majority of the Amhara from the majority of the Oromo individuals; in addition, within each ethnic group, the HA and LA individuals tend to cluster separately (Figure S3. Accordingly, the mean F_ST_ between LA Amhara and Oromo is very low (0.0098), though it is higher than the mean F_ST_ between LA and HA populations within the same ethnic group (HA *vs.* LA Amhara: F_ST_=0.0047; HA *vs*. LA Oromo high F_ST_=0.0074). The STRUCTURE analysis did not reveal detectable differences between the HA and LA populations (10^6^ iterations; following a burn-in period of 30,000 iterations; k=3) (Figure S4. However, when we compared the F_ST_ distribution between HA and LA populations (Amhara or Oromo) to the distribution obtained by permuting the altitude labels (*i.e*. under random mating for HA and LA populations within each ethnic group) we detected a significant excess of high F_ST_ values, indicating that some population structure between altitudes does exist (Figure S5). In order to test for an excess of allele frequency divergence between populations, the observed distribution of F_ST_ was compared to a null distribution obtained by permuting the population labels 100 times, and for each comparison, a different permuted F_ST_ distribution was created. Permutations were used to estimate the 95% confidence interval.

REFERENCES

1. Hancock AM, Witonsky DB, Ehler E, Alkorta-Aranburu G, Beall C, et al. (2010) Colloquium paper: human adaptations to diet, subsistence, and ecoregion are due to subtle shifts in allele frequency. Proc Natl Acad Sci U S A 107 Suppl 2: 8924-8930.

2. Pritchard JK, Stephens M, Donnelly P (2000) Inference of population structure using multilocus genotype data. Genetics 155: 945-959.

3. Li JZ, Absher DM, Tang H, Southwick AM, Casto AM, et al. (2008) Worldwide human relationships inferred from genome-wide patterns of variation. Science 319: 1100-1104.
