# Supplementary material for: The Genetic Architecture of Adaptations to High Altitude in Ethiopia
Source: PLoS Genet. 2012 Dec 6;8(12):e1003110. doi: 10.1371/journal.pgen.1003110 (PMC3516565; doi:10.1371/journal.pgen.1003110)
Supplement: Text S3 — Phenotypic variation in Amhara and Oromo. (DOCX) [file pgen.1003110.s052.docx]

**Text S3. Phenotypic variation in Amhara and Oromo**

260 unrelated people provided the phenotype and genotype data presented here. HA Amhara were sampled in 1995 (n=47) and 2005 (n=65) and the LA Amhara in 2005-6 (n=59). All Oromo were sampled in 2007 (n=88). These unrelated people were identified from a larger sample of partially related individuals.

In the Semien Mountain area, two models of Criticare oximeter were used, the Criticare Model 503 was used for 152 people and the SpO2 was used for 150 people. Twenty-five people were measured using both instruments and the average difference (SpO2 minus 503) was =0.031 + 2 (SD) %. One observation was just outside the limits of agreement of the two models (+ 2 SDs). The Model 503 values were used as the O_2_ sat measured by Criticare oximeter for the 127 who did not have SpO2 measurements.

Table S1 summarizes height, weight, body mass index (BMI), and pulse. Considering altitude differences, HA Amhara native men and women were shorter, lighter and had lower BMI than their lowland Amhara counterparts. HA Oromo native men had higher BMI than their lowland Oromo counterparts although there were no significant differences in height or weight. HA Oromo native women were shorter but did not differ in weight or BMI from their lowland counterparts. Considering ethnic differences within altitude zones, HA Amhara men and women were shorter, lighter and had lower BMI than Oromo. LA Amhara men were heavier and had higher BMIs. Amhara lowland women did not differ from Oromo lowland women in these characteristics although there was a trend toward shorter height.

Table S2 summarizes the three HA adaptation phenotypes, hemoglobin (Hb) concentration, percent of oxygen saturation (O_2_ sat) of Hb and calculated arterial oxygen content (AOC). Arterial oxygen content in ml O_2_/dL was calculated as 1.39*(Hb*O_2_ sat)/100 [[1](#_ENREF_1)]. Phenotypes were assessed using the same equipment and protocols in all areas [[2](#_ENREF_2)]. To remove many possible confounding factors that could have added spurious variation to the phenotypes, the analyses were confined to healthy people (based on self-report and a review of systems by an Ethiopian physician), who were free of respiratory symptoms, not hypertensive, not pregnant, had not delivered an infant in the past year and had not visited at another altitude (below 2500m for highlanders or above 2500m for lowlanders) in the past six months. Samples collected in 2005, 2006 and 2007 were also screened for normal lung function and for infection with all four species of human-associated malaria [[3](#_ENREF_3)]. Furthermore, because poor iron status could limit the Hb response to HA hypoxia, the analyses were limited to those with normal iron status. Iron deficiency in the 1995 sample was identified on the bases of zinc erythrocyte protoporphyrin > 70 gm/dL, plasma ferritin concentration <12 ng/ml or transferrin receptor concentration <8.3 g/L [[2](#_ENREF_2)]. Iron deficiency in the samples collected later was identified on the bases of body iron stores calculated using the log of the ratio of transferrin receptor to ferritin concentration [[4](#_ENREF_4)]. If an individual were missing data for any of these variables, then his or her Hb concentration data were not analyzed for association with genetic variants. Stringent criteria for phenotypes improve the likelihood of finding phenotypic variation associated with health, normal genetic variation and add confidence to findings of a lack of association.

With respect to altitude differences, Hb was significantly higher among all four HA samples compared with the age-sex groups at LA but size of the effect was smaller among the Amhara (Table S2 and Figure S6. Similarly, the percent of O_2_ sat was lower among all four HA samples compared with the age-sex groups at LA, but the size of the effect was smaller among the Amhara (Table S2 and Figure S6. The altitude sub-samples did not differ from one another in calculated AOC. With respect to ethnic group differences within altitude zones, Amhara had lower Hb concentrations at both HA and LA (Table S2 and Figure S6 show that the differences were more than one gm/dL of Hb and more than one standard deviation). In contrast, Amhara had higher percent of O_2_ sat at HA than their Oromo counterparts. The result was similar for AOC among highlanders but a trend toward lower AOC among LA Amhara males that was statistically significant among Amhara females was observed.

In summary, there were altitude and ethnic group differences in phenotypes with the Oromo showing a larger altitude response than the Amhara.

REFERENCES

1. West JB (1985) Respiratory Physiology - The Essentials. Baltimore, MD.: Williams &Wilkins

2. Beall CM, Decker MJ, Brittenham GM, Kushner I, Gebremedhin A, et al. (2002) An Ethiopian pattern of human adaptation to high-altitude hypoxia. Proc Natl Acad Sci U S A 99: 17215-17218.

3. Hoit BD, Dalton ND, Gebremedhin A, Janocha A, Zimmerman PA, et al. (2011) Elevated pulmonary artery pressure among Amhara highlanders in Ethiopia. American journal of human biology 23: 168-176.

4. Cook JD, Flowers CH, Skikne BS (2003) The quantitative assessment of body iron. Blood 101: 3359-3364.
