# Supplementary material for: The Genetic Architecture of Adaptations to High Altitude in Ethiopia
Source: PLoS Genet. 2012 Dec 6;8(12):e1003110. doi: 10.1371/journal.pgen.1003110 (PMC3516565; doi:10.1371/journal.pgen.1003110)
Supplement: Text S4 — Comparing the genetic architecture of Hb levels between Ethiopian and Tibetans. (DOCX) [file pgen.1003110.s053.docx]

**Text S4. Comparing the genetic architecture of Hb levels between Ethiopian and Tibetans**

To compare the genetic architecture of Hb levels between Ethiopians and Tibetans [[1](#_ENREF_1),[2](#_ENREF_2),[3](#_ENREF_3)], we performed association tests and power analyses at three levels: 1) the SNPs in *EPAS1* and *EGLN1* associated with the trait in Tibetans, 2) all the SNPs within the same genes, and 3) all the SNPs within all the genes in the same pathway, i.e. response to hypoxia.

The *EPAS1* and *EGLN1* SNPs that were previously associated with variation in Hb levels in Tibetans have effect sizes of 0.8 g/dL and 1.7 g/dL, respectively [[1](#_ENREF_1),[2](#_ENREF_2)]. Of these SNPs, we considered those that were genotyped or imputed with greater than 90% accuracy in our Ethiopian data. Because the lack of association in the Ethiopians could be due to incomplete power, we calculated the probability of observing a significant association between SNP genotype and Hb levels in our data, assuming that the β coefficient for each SNP is as high as or higher than that observed in Tibetans and using the corresponding MAF in the Ethiopians. As shown in Table 1, we have complete or nearly complete power to detect a genotype-phenotype association in our Ethiopian samples. This suggests that either the SNPs associated with variation in Hb levels in the Tibetans do not make a contribution in Ethiopians or if they do their phenotypic effect is smaller in Ethiopia. However, given their corresponding MAFs in Amhara, Oromo and Ethiopian, we have nearly complete power to detect a significant association even if the phenotypic effect was half as large as that observed in the Tibetan studies (Table S21).

Next, we considered all SNPs within 10kb of the *EPAS1* gene and repeated this analysis applying a Bonferroni correction for the number of tests performed (*i.e*., 72 SNPs). We find that we have greater than 95% power to detect a SNP with an effect size of at least 0.8 g/dL Hb concentration if the MAF in Ethiopia (Figure 3A) is greater than 10% (see Figure S16 for Amhara and Oromo). When we performed the same analysis for SNPs within 10kb of the *EGLN1* gene (38 SNPs), we find that we had 100% power to detect SNPs with effect size of at least 1.7 g/dL Hb concentration if the SNP MAF in Ethiopia is greater than 5% (Figure 3B and Figure S16 for Oromo and Amhara). Because none of the *EPAS1* or *EGLN1* SNPs was significantly associated with Hb levels in Ethiopians after multiple test correction, this analysis suggests that genes shown to contribute to variation in Hb levels in Tibetans either do not influence variation in the Ethiopian populations or if they do, their effect sizes are lower than those reported for the Tibetans.

Finally, we considered the SNPs within 10kb of the candidate genes in the “Response to Hypoxia” GO category (26 genes). Because of the larger number of SNPs tested, this analysis has a relatively high multiple testing burden. Nonetheless, we find that we have greater than 80% power to detect a SNP significantly associated with Hb levels and effect size 0.8g/dL if its MAF is at least 20% and 100% power if its effect size is 1.7g/dL Hb (Figure 3C and Figure S16 for Oromo and Amhara).

REFERENCES

1. Simonson TS, Yang Y, Huff CD, Yun H, Qin G, et al. (2010) Genetic evidence for high-altitude adaptation in Tibet. Science 329: 72-75.

2. Beall CM, Cavalleri GL, Deng L, Elston RC, Gao Y, et al. (2010) Natural selection on EPAS1 (HIF2alpha) associated with low hemoglobin concentration in Tibetan highlanders. Proc Natl Acad Sci U S A 107: 11459-11464.

3. Yi X, Liang Y, Huerta-Sanchez E, Jin X, Cuo ZX, et al. (2010) Sequencing of 50 human exomes reveals adaptation to high altitude. Science 329: 75-78.
